# Via Air or Rhizosphere: The Phytotoxicity of *Nepeta* Essential Oils and *Malus* Dihydrochalcones

**DOI:** 10.3390/plants14050701

**Published:** 2025-02-25

**Authors:** Slavica Dmitrović, Jasmina Nestorović Živković, Dijana Smailagić, Milena Trajković, Nevena Banjac, Slavica Ninković, Mariana Stanišić

**Affiliations:** Institute for Biological Research “Siniša Stanković”, National Institute of the Republic of Serbia, University of Belgrade, Bulevar Despota Stefana 142, 11108 Belgrade, Serbia; jasmina.nestorovic@ibiss.bg.ac.rs (J.N.Ž.); dijana.smailagic@ibiss.bg.ac.rs (D.S.); milena.lojic@ibiss.bg.ac.rs (M.T.); mitic.nevena@ibiss.bg.ac.rs (N.B.); slavica@ibiss.bg.ac.rs (S.N.)

**Keywords:** allelopathy, bioherbicides, *Malus* dihydrochalcones, essential oil, *Nepeta*, nepetalactone, phytotoxicity

## Abstract

Many specialized metabolites found in plants have significant potential for developing environmentally friendly weed management solutions. This review focuses on the phytotoxic effects of volatile terpenes and phenolic compounds, particularly nepetalactone, an iridoid monoterpenoid from *Nepeta* species, and phloretin, a dihydrochalcone predominantly found in the genus *Malus*. We highlight current findings on their herbicidal effects, including morphological, physiological, and biochemical responses in target plants. These results underscore their potential for developing sustainable herbicides that could control weeds with minimal environmental impact. We also discuss their soil persistence and methods to enhance their solubility, chemical stability, and bioavailability. Additionally, the possible effects on non-target organisms, such as pollinators, non-pollinating insects, and soil microbiota, are considered. However, further research and a deeper understanding of their long-term ecological impact, along with a resistance development risk assessment, is essential for the potential development of bioherbicides that could be applied in sustainable weed management practices.

## 1. Introduction

Secondary metabolites are important bioactive compounds produced by plants and are indispensable for their survival and ecological interactions. Unlike primary metabolites, which are essential for growth and development, these specialized molecules play a crucial role in protecting plants from herbivores, pathogens, and adverse environmental conditions. They also contribute to reproductive success by attracting pollinators and facilitating complex communication within plant communities. The vast diversity of these compounds is astounding, with estimates suggesting the existence of over one million unique secondary metabolites in the plant kingdom, though only around 200,000 have been identified and studied to date [1].

The production of specialized metabolites with phytotoxic properties (allelochemicals) offers advantages, such as establishing dominance in a plant’s natural habitat by outcompeting neighboring species for light, water, and essential nutrients. Such a scenario is achieved by inhibiting the growth, development, and reproduction of nearby plant species through the release of allelochemicals into the environment. This natural phenomenon, known as allelopathy [2], provides these plants with a significant evolutionary advantage. Allelochemicals (prevalently phenolics, terpenoids, and nitrogen-containing compounds [3,4,5,6]) are very diverse in their chemical nature, which allows them to target the same physiological processes in competing species through different molecular approaches and mechanisms. Plants can release such phytochemicals into their environment through various mechanisms, including root exudation, leaching, volatilization, and decomposition of plant residues. These processes facilitate the transfer of allelochemicals into the surrounding soil, water, and air, where they can influence the growth and development of neighboring plants and/or microorganisms [7]. Different cellular processes, such as the alteration of cellular micro- and ultra-structure, disruption of cell division and elongation, and alteration of membrane permeability and the antioxidant system, are often targeted by various allelochemicals. From a broader perspective, it could be said that allelochemicals affect all major physiological processes in plants such as growth, photosynthesis, respiration, hormone homeostasis, uptake of ions, metabolism, and the synthesis of proteins and nucleic acids [8,9]. The highly diverse mechanisms of action that allelochemicals employ to influence key physiological processes in competing species can be leveraged for the development of bioherbicides with novel modes of action, addressing various challenges in conventional agricultural practices [10,11].

In the era of widespread reliance on synthetic herbicides, the environmental consequences are becoming increasingly apparent, including soil and water contamination and broader ecological degradation. As a result, there is a growing need for sustainable alternatives that minimize environmental harm and reduce synthetic herbicide dependency [12,13]. The development of bioherbicides mostly relies on the utilization of living organisms or natural metabolites produced during their growth and development [14]. The bioherbicide market has been developing since 1981, mainly using microbial ingredients [15]. However, limited markets and technical difficulties associated with mass production were the two main reasons that reduced the commercialization of such bioherbicides [15,16,17,18]. In 2016, only thirteen bioherbicides were registered, of which nine were of mycological origin, three of bacteriological origin, and only one of plant origin [19]. More recently, attempts to derive bioherbicides from some viruses have also been reported [20]. Of all biopesticides approved by the US Environmental Protection Agency, only 7% are bioherbicides [21]. As scientific progress continues in addressing the weed problem, the definition of the term ’bioherbicide’, which once included only living microorganisms and their products, is evolving. It is now known that phytochemicals, such as allelochemicals and their synthetic derivatives, can be used as active ingredients in bioherbicides or botanical-based synthetic herbicides [22]. However, obtaining plant-derived bioherbicides is a challenging endeavor. Analyzing the chemical composition of released allelochemicals or crude extracts from allelopathic species may reveal new compounds with phytotoxic effects. Research into their mode of action is the crucial first step in evaluating them as potential bioherbicides. Furthermore, allelochemicals can be used as a foundation for synthesizing synthetic derivatives with enhanced phytotoxicity or stability, making them suitable as botanical-based synthetic herbicides or key components of herbicidal formulations. However, the production of bioherbicides on a commercial scale is hampered by numerous issues concerning stability, formulation, environmental impact, weed resistance, regulatory approvals, cost-effectiveness, and scalability [23]. Despite various challenges in the design and formulation of bioherbicides, some plant-derived herbicidal compounds have been commercially accepted. For example, the commercial herbicide cinmethylin, an analog of the natural phytotoxin 1,4-cineole, was discovered and developed by the American company Shell Chemicals [24]. Cinmethylin is a moderately effective growth inhibitor used to control monocotyledonous weeds [25]. The mechanism of action of cinmethylin is based on the inhibition of acyl-ACP thioesterases and plant fatty acid biosynthesis [26]. Triketone herbicides are derivatives of the phytotoxin leptospermone, which is produced by some members of the Myrtaceae family, such as *Callistemon citrinus* and *Leptospermum scoparium* [27]. The molecular target of triketone herbicides is *p*-hydroxyphenylpyruvate dioxygenase, whose inhibition indirectly reduces phytoene desaturase activity, further decreasing carotenoid levels and resulting in the destabilization of the photosynthetic apparatus [28]. Recently, work has been conducted on the formulation of natural herbicides from essential oils (EOs), resulting in six such preparations being registered in the USA [29].

A major challenge in transitioning to the use of bioherbicides is the availability of suitable formulations and application methods that ensure that the biotic active ingredient is evenly distributed in the target area without wastage or overapplication, which could increase treatment costs and the risk of side effects. One of the newer approaches to improving bioherbicide formulations is through the use of nanotechnology [30]. The method of nanoencapsulation is especially suitable for small plant-derived molecules as it enhances their stability, effectiveness, and bioavailability, as well as enabling precise targeting and controlled release [30].

**Figure 1:**
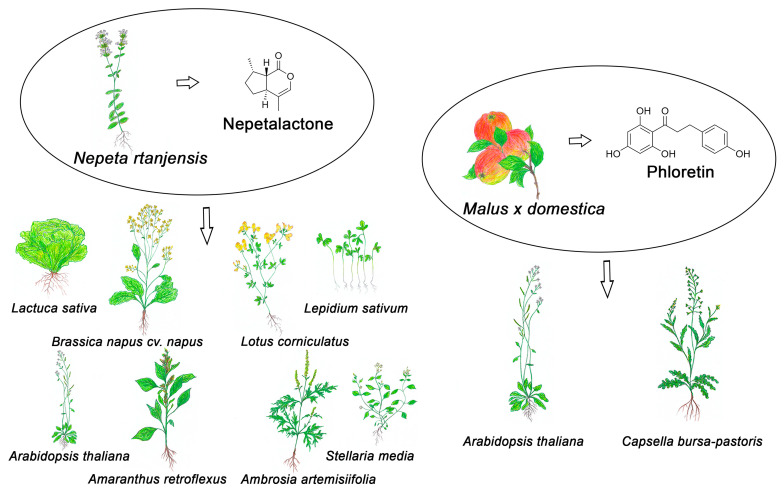
Cultivated and weed species used to assess the phytotoxic effects of nepetalactone or phloretin.

In light of the aforementioned issues and the increasing demand for environmentally friendly herbicides, our research group has focused on investigating the phytotoxic effects of specialized plant metabolites on selected model and target plants, with the goal of identifying potential candidates for the development of future bioherbicides. One direction of our research involves soil-exuded allelochemicals produced by plants such as the apple (*Malus* × *domestica* Borkh.) and its most prevalent specialized metabolite, phloretin [31,32,33,34], while the other focuses on the effects of volatile organic compounds (VOCs), particularly those emitted from EOs from *Nepeta* species [35,36,37,38,39,40,41]. The investigation of phloretin phytotoxicity is based on the model species thale cress (*Arabidopsis thaliana* (L.) Heynh.) ecotype Columbia, and shepherd’s purse (*Capsella bursa-pastoris* (L.) Medik), a weed species from the Brassicaceae family. The target plants included in the investigation on *Nepeta* EOs are weed species that cause considerable yield losses in crops due to being resistant to commercial herbicides. These include common ragweed (*Ambrosia artemisiifolia* L.), redroot ragweed (*Amaranthus retroflexus* L.), and chickweed (*Stellaria media* (L.) Vill.) (International Herbicide Resistance—Weed Database, https://www.weedscience.org, accessed on 4 January 2025), while the selected fast-growing crops included lettuce (*Lactuca sativa* L.), oilseed rape (*Brassica napus* L. cv. *napus*), garden cress (*Lepidium sativum* L.), and bird’s-foot trefoil (*Lotus corniculatus* L.) (Figure 1).

Here, we present recent findings on the phytotoxic effects of volatile terpenes and phenolic compounds, particularly nepetalactone (NL), an iridoid monoterpenoid from *Nepeta* species, and phloretin, a dihydrochalcone (DHC) predominantly found in the genus *Malus*, focusing on morphological, physiological, and biochemical responses in target plants. These findings highlight their potential for developing sustainable, eco-friendly herbicides that could effectively manage weeds while minimizing environmental impact and reducing reliance on synthetic chemicals. Additionally, we discuss available data on their persistence in soil and strategies to enhance their solubility, chemical stability, and bioavailability. Potential effects on non-target organisms, including pollinators, non-pollinating insects, and soil microbiota, are also considered.

## 2. Phytotoxic Effects of Volatile Terpenes from the Lamiaceae Family

Terpenes are a widespread group of specialized metabolites, with hydrocarbon structures consisting of five carbon units—isoprene units (CH2=C(CH3)CH=CH2)—that can be converted into cyclic forms [42]. Two key C_5_ isoprenoid precursors, isopentenyl diphosphate (IPP) and its double bond isomer dimethylallyl diphosphate (DMAPP), play a central role in terpene biosynthesis [43,44]. In plants, these precursors are synthesized via two different metabolic pathways: the cytosolic mevalonate (MVA) pathway, which originated from acetyl-CoA, and the plastidial 2-C-methyl-D-erythritol 4-phosphate pathway (MEP), which is derived from pyruvate and glyceraldehyde 3-phosphate [45,46]. Through a series of enzyme-directed reactions involving rearrangements, elongations, and cyclizations, IPP and DMAPP are converted into different classes of terpenes [47,48,49].

Terpenoids are chemically modified terpenes that often contain oxygenated groups such as hydroxyl (-OH), carboxyl (-COOH), ketone (-C=O), or aldehyde (-CHO) groups. These modifications provide them with greater chemical diversity and various bioactive properties [50]. According to their isoprene units, terpenes are divided into hemiterpenes (one unit, C_5_), monoterpenes (two units, C_10_), sesquiterpenes (three units, C_15_), diterpenes (four units, C_20_), triterpenes (six units, C_30_), and tetraterpenes (eight units, C_40_) [51]. Geranyl pyrophosphate synthase (GPPS) and farnesyl pyrophosphate synthase (FPPS) catalyze the production of geranyl pyrophosphate (GPP), a precursor for C_10_ monoterpenes, and farnesyl pyrophosphate (FPP), the precursor for C_15_ sesquiterpenes. Similarly, geranylgeranyl pyrophosphate synthase (GGPPS) and farnesyl geranyl pyrophosphate synthase (FGPPS) facilitate the formation of geranylgeranyl pyrophosphate (GGPP), a precursor for C_20_ diterpenes, and farnesyl geranyl pyrophosphate (FGPP), which leads to C_25_ sesquiterpenes. These intermediates (GPP, FPP, GGPP, and FGPP) serve as substrates for the terpene synthase enzyme family, which catalyzes a series of cyclization and rearrangement reactions that diversify the structural and functional properties of terpenoids and contribute to the wide range of terpenoid classes in nature [1,52,53].

**Figure 2:**
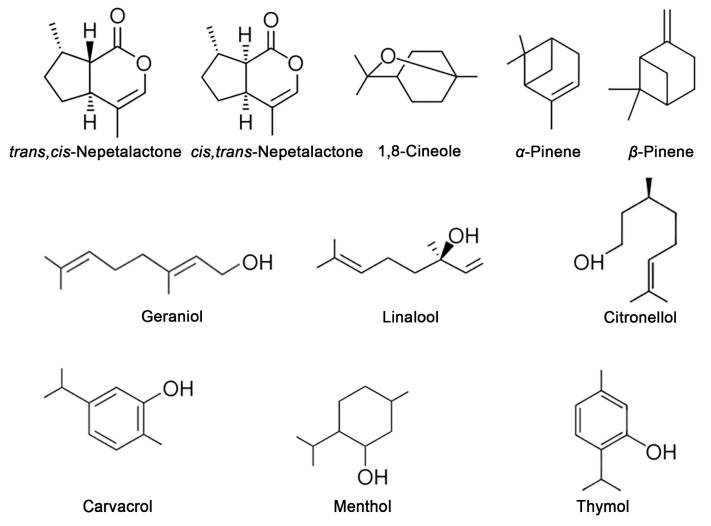
The chemical structures of bioactive monoterpenes from essential oils of the Lamiaceae family.

Monoterpenes and sesquiterpenes, along with their oxygenated derivatives, are VOCs that significantly contribute to the distinctive aroma and olfactory profile of plants. Species within the Lamiaceae family are especially abundant in these compounds, which are integral to the characteristic fragrances exhibited by plants in this family. The Lamiaceae family comprises more than 250 genera and about 7000 species [54], which are widely used in the pharmaceutic, cosmetic, and food industries due to their antioxidative, antibacterial, antifungal, antiviral, cardioprotective, anti-inflammatory, antidiabetic, and anticancer properties [55,56,57,58,59,60,61]. In addition to numerous pharmacological and biological activities, species from the Lamiaceae family also exhibit strong phytotoxic effects against various target plants, which are attributed to the monoterpenes and their derivatives contained in the EOs (Figure 2) [62,63,64,65]. The monoterpenes have been described as the main constituents of the EOs of eight Mediterranean species from the Lamiaceae family (*Hyssopus officinalis* L., *Lavandula angustifolia* Mill., *Majorana hortensis* Moench., *Melissa officinalis* L., *Ocimum basilicum* L., *Origanum vulgare* L., *Salvia officinalis* L., and *Thymus vulgaris* L.). The EOs of *M. officinalis* and *T. vulgaris* showed a strong phytotoxic effect on the seed germination and growth of the radicles of *Raphanus sativus* L., *Lactuca sativa* L., and *Lepidium sativum* L. via different mechanisms [66]. The EO of *Mentha × piperita* L., which is rich in menthone, menthol, and menthofuran, has been shown to strongly inhibit seed germination and seedling growth in three weed species: field bindweed (*Convolvulus arvensis* L.), purslane (*Portulaca oleracea* L.), and jungle rice (*Echinochloa colona* L.), as well as two horticultural crops, tomato (*Lycopersicon esculentum* Mill.) and radish (*Raphanus sativus* L.) [67]. The monoterpenoids carvacrol and thymol, found in the EO of *Origanum acutidens* L., completely inhibited seed germination and seedling growth in three weed species: *Amaranthus retroflexus* L., *Chenopodium album* L., and *Rumex crispus* L. [68]. The main constituents of *Salvia sclarea* L. EO, the monoterpenes L-linalool, linalyl acetate, *α*-terpineol, and geraniol showed a strong inhibitory effect on the seed germination of *L. sativum, L. sativa,* and *P. oleracea* [69]. Casella et al. [70] investigated the phytotoxic effects of thirteen EOs derived from six wild Lamiaceae species: *Clinopodium suaveolens* L., *Satureja montana* L. subsp. *montana*, *Thymbra capitata* (L.) Cav., *Salvia fruticosa* Mill. subsp. *thomasii*, *Satureja cuneifolia* Ten., and *Thymus spinulosus* Ten. The study assessed their impact on the seed germination of *L. sativum* and *Phelipanche ramose* L. seeds, the elongation of *Solanum lycopersicum* L. radicles, and the chlorophyll (Chl) and carotenoid content in *C. album* leaves. Notably, the EO from *T. capitata* inhibited *L. sativum* seed germination and significantly reduced Chl and carotenoid levels in *C. album*. Additionally, the EO from *T. spinulosus* suppressed *S. lycopersicum* radicle elongation. The observed phytotoxic effects were associated with key EO components such as thymol and *p*-cymene, highlighting their potential application as pre-emergence herbicides. Tudora et al. [71] investigated the phytotoxic effects of *Hyssopus officinalis* L. EO on the seed germination and seedling growth of two weed species (*Setaria viridis* L. and *Sorghum halepense* (L.) Pers.) and two vegetable crops (*L. sativa* and *Spinacia oleracea* L.). The EO, characterized by high levels of *cis*-pinocamphone, *trans*-pinocamphone, and *β*-pinene, exhibited species- and concentration-dependent effects, either inhibiting or promoting seed germination and growth. Cineole and citronellol, commonly found in plants of the Lamiaceae family, significantly inhibited seed germination, reduced seed germination rates, suppressed seedling growth, and reduced the Chl content and respiratory activity of a noxious weed—*Ageratum conyzoides* L. [72]. Furthermore, the EOs of *O. vulgare* ssp. *hirtum*, rich in monoterpenes such as carvacrol, thymol, and ocymene, significantly reduced *A. thaliana* growth, causing leaf chlorosis and metabolic disruptions, especially nitrogen assimilation, through glutamine metabolism, which led to toxic ammonia accumulation, oxidative stress, reduced photosynthetic efficiency, and photorespiration [73]. Romagni et al. [74] revealed the different phytotoxic effects of two volatile monoterpene isomers, 1,4-cineole and 1,8-cineole, which have the same molecular formula (C_10_H_18_O) but differ in the position of their oxygen bridges. Despite their structural similarity, the isomers showed different biological activities in two tested weeds, the monocot *Echinochloa crus-galli* (L.) P.Beauv. and the dicot *Cassia obtusifolia* L. 1,4-Cineole inhibited root and shoot growth, led to morphological distortions, and caused significantly greater photosynthetic stress, while 1,8-cineole primarily reduced root growth and seed germination rates. Analysis of the mitotic index revealed that 1,8-cineole suppressed all stages of mitosis, while 1,4-cineole only caused a reduction in the prophase stage compared to the controls. These findings highlight the crucial role of molecular conformation in determining the phytotoxic effect of bioactive compounds and emphasize the importance of considering molecular structure when evaluating the potential roles and synergistic/antagonistic interactions of compounds in plants. Moreover, it underscores the need for in-depth studies on the mechanisms of action of different stereoisomers on seed germination and the growth of weeds. Such research is essential for advancing eco-friendly agricultural practices, offering a promising alternative to synthetic herbicides and contributing to the development of sustainable, natural herbicides for effective weed management.

### 2.1. Phytotoxic Effects of Monoterpenes from the Genus Nepeta

The genus *Nepeta*, (subfamily Nepetoideae, tribe Mentheae), commonly known as catmint, is one of the largest genera within the Lamiaceae family and comprises about 300 species [75,76]. It includes perennial herbaceous plants that are mainly distributed in Central and Southern Europe, the Middle East, Central and South Asia, and parts of Africa [77,78] (Plants of the World online 2024—https://powo.science.kew.org, accessed on 4 January 2025). *Nepeta* species are widely recognized in traditional medicine for their multiple therapeutic properties [78,79], but also as an effective natural insect repellent against mosquitoes, cockroaches, flies, and termites and as a powerful attractant for cats [80,81]. Field studies have identified inhibition zones around certain *Nepeta* species, where competing vegetation is notably absent. This phenomenon strongly suggests that *Nepeta* species possess allelopathic properties [82]. Studies on *Nepeta* species have identified monoterpenoids, sesquiterpenoids, diterpenoids, triterpenoids, flavonoids, and phenolic compounds as major specialized metabolites that could play a role in allelopathic interactions [80]. The majority of the biological and pharmacological activities in *Nepeta* species are attributed to their iridoid compounds [58]. Iridoids, a group of cyclopentane pyran monoterpenoids, are predominantly found as glycosides, with glucose often linked to their C-1 or C-11 positions. Of the over 3000 known iridoids, only about 80 are aglycones, a notable proportion of which are exclusively found in the genus *Nepeta*. Nepetalactone, a volatile iridoid aglycone, is considered the most important bioactive compound in the EOs of iridoid-rich *Nepeta* species and occurs in eight stereoisomeric forms, including four diastereoisomers and the corresponding enantiomers [76]. Although these NL isomers differ only in the orientation of a single chemical bond, they exhibit different biological activities [83]. With a few exceptions, only the *7S* diastereoisomers have been identified in nature. The yield and the chemical composition of EOs vary in different *Nepeta* species depending on genetic and environmental factors [84].

The biosynthesis of NL in *Nepeta* begins with GPP, a precursor derived from the MEP pathway, as is the case for all monoterpenes. GPP is converted into geraniol by the enzyme geraniol synthase (GES) [85]. Geraniol is then hydroxylated by the cytochrome P450 enzyme geraniol 8-hydroxylase (G8H) [86] and further oxidized by hydroxygeraniol oxidase (HGO) [87] to form 8-oxogeranial. The homologs of GES, G8H, and HGO in *Nepeta* are highly similar to those found in other iridoid-producing plants [86]. Subsequently, the enzyme iridoid synthase (ISY) catalyzes the 1,4-reduction of 8-oxogeranial, producing 8-oxocitronellyl enol, which spontaneously cyclizes to predominantly form the *cis,trans-* stereoisomer of NL [88]. The discovery of stereochemical isomers of NL in *Nepeta* (*cis,trans-*, *trans,cis-*, and *cis,cis-*) revealed that while the ISY product 8-oxo-6S-citronellyl enol can non-enzymatically cyclize into the *cis,trans-*NL stereoisomer, cyclization in the plant is facilitated by specific enzymes that regulate the formation of distinct NL stereoisomers [89]. Three stereoselective cyclase enzymes were identified in the trichomes of *Nepeta cataria* and *N. mussinii* [86,89]. These enzymes, which resemble short-chain dehydrogenase-like (SLN-like) enzymes, including NAD-dependent nepetalactol-related short-chain dehydrogenase/reductases (NEPSs) NEPS3 and NEPS4, catalyze the cyclization of 8-oxocitronellyl enol into the *cis,cis-* and *trans,cis*-NL isomers. Coding sequences for NrNEPS1, identified in the *N. rtanjensis* transcriptome [90], and NcNEPS5 from *N. cataria* [86] were also characterized.

Earlier studies on *Nepeta* species have revealed organ-specific expression patterns of genes involved in iridoid biosynthesis [86,91,92]. Closed buds and open flowers of *N. cataria* and *N. mussinii* exhibited higher transcript levels of *NEPS*, *G8H*, and *ISY* genes compared to mature leaves [86]. Among the *NEPS* genes, *NEPS1* showed the highest expression in the open flowers and closed buds of *N. cataria*, while *NEPS5* transcripts were most abundant in the closed buds of *N. mussinii* [86]. In *N. nuda*, genes responsible for the steps directly preceding the formation of nepetalactol and NL (*NnNEPS1*, *NnNEPS2*, *NnNEPS3*, and *NnMLPL*) showed significantly higher expression levels in inflorescences than in leaves [84]. Additionally, a major latex protein-like (MLPL) enzyme catalyzes the formation of the *cis,trans-* isomer [86]. Enzymes such as NEPS, NEPS5, and NEPS1 subsequently oxidize these nepetalactols into NL [86,89]. In species that do not have detectable amounts of iridoids in their leaves, such as *N. nervosa*, there is likely a silent biosynthetic platform for iridoids [92]. It is assumed that some components are missing in the iridoid biosynthetic pathway of *N. nervosa*, which impairs the biosynthesis of iridoids. This includes the absence of GES-like activity due to gene silencing, leading to the absence of both iridoid aglycones and iridoid glycosides, as well as the essential role of other NEPS and MLPL enzymes, in addition to NnNEPS1 and NnMLPL, in the biosynthesis of iridoid aglycones [92].

It has been well-documented that EOs of *Nepeta* species have shown strong inhibitory effects on seed germination and seedling growth in different target species. Table 1 provides a detailed summary of the phytotoxic activity of the EOs of different *Nepeta* species, highlighting the dominant compounds detected in the EOs, the plant species tested, the experimental design with the doses of the EOs or dominant compounds used, and the phytotoxic activities observed. Dmitrović et al. [40] showed that treatment with *N. rtanjensis* EO affected various physiological and metabolic processes in *A. thaliana*. Kordali et al. [93] reported that *N. meyeri* EO completely suppressed the seed germination of four weed species: *Amaranthus retroflexus* L., *Chenopodium album* L., *Cirsium arvense* (L.) Scop., and *Sinapis arvensis* L. The most sensitive weed to *N. mayeri* EO spraying was *A. retroflexus*. Saharkhiz et al. [94] demonstrated that the weed *Taraxacum officinale* was the most affected by *N. cataria* EO, while the crop species *Lepidium sativum* was the most tolerant. In addition, Mutlu et al. [95] found that *N. meyeri* EO inhibited seed germination and altered the antioxidative activity of seven weed species, with *Bromus danthoniae* Trin., *Bromus intermedius* Guss., and *Lactuca serriola* L. being the most perceptive. Furthermore, Dyanat et al. [96] examined the phytotoxic properties of EOs from *N. glocephalata* Rech.f. and *N. ispahanica* Boiss. on four weed species, finding that *A. retroflexus* and *C. album* were the most affected. In addition, Diyanat et al. [97] investigated the bioherbicidal activity of EOs from *Nepeta menthoides* Boiss & Buhse, *N. mahanensis* Jamzad & Simmonds, *N. elymaitica* Bornm, and *N. binaludensis* Jamzad on two weed species: wild mustard (*Sinapis arvensis*) and winter wild oat (*Avena ludoviciana* Dur.). Although wild mustard was more sensitive to the *Nepeta* EOs than winter wild oat, the study concluded that *Nepeta* EOs have phytotoxic effects and could be used as a bioherbicide to control both weeds tested. The diverse nature of monoterpenoids, coupled with their wide range of biological activities and proven phytotoxic effects, highlights their significant potential for further investigation and the development of monoterpenoid-based formulations as effective bioherbicides in sustainable agriculture.

### 2.2. The Biological Activity of Essential Oils Is Determined by the Stereochemistry of Nepetalactones

In vitro culture provides sterile, controlled conditions that enable assessing the direct effects of various components on plant growth and development [103]. As far as we know, Dmitrović et al. [37] first established ragweed (*Ambrosia artemisiifolia*) shoot cultures in vitro and subsequently investigated the phytotoxic effects of *N. rtanjensis* and *N. cataria* EOs. *N. rtanjensis* is an endemic species of the genus *Nepeta*, restricted to small natural populations in the Rtanj mountain in south-eastern Serbia [104]. To support its conservation and explore its commercial potential, advanced propagation systems have been introduced, including in vitro micropropagation as well as vegetative propagation in greenhouse and field conditions [105]. On the other hand, *N. cataria* is widely distributed in the Serbian flora. The primary components of the *N. rtanjensis* EO (*Nr*EO) and *N. cataria* EO (*Nc*EO) are distinct stereoisomers of NL. *Nr*EO contains the *trans,cis*- isomer as the dominant compound, while *Nc*EO is characterized by the *cis,trans*- isomer of NL. Other monoterpenoids such as *α*- and *β*-pinene and sesquiterpenoids were present in lower amounts [37].

The NLs in the *Nr*EO and *Nc*EO inhibited the growth of ragweed shoots and roots and caused shoot discoloration, probably due to the degradation of Chl or reduced synthesis [37]. These results align with earlier studies [72] suggesting that volatile monoterpenes can negatively impact photosynthesis in goat weed (*Ageratum conyzoides* L.). In addition, Verdeguer et al. [29] reviewed that EOs negatively impact the photosynthetic system and energy metabolism of target plants. In addition, Dmitrović et al. [37] demonstrated that both *cis,trans*- and *trans,cis*-NLs inhibit shoot growth, while *trans,cis*-NL impairs root growth to a greater extent. In addition, Singh et al. [106] demonstrated that *α*-pinene inhibited the radicle growth of *Cassia occidentalis* L., *Amaranthus viridis* L., *Triticum aestivum* L., *Pisum sativum* L., and *Cicer arietinum* L., while Chowhan et al. [107] tested *β*-pinene for its phytotoxic effects against two grassy weeds (*Phalaris minor* Retz. and *Echinochloa crus-galli* (L.) P.Beauv.) and a broadleaf weed (*Cassia occidentalis*). *β*-Pinene was found to inhibit seed germination and root and shoot growth in a dose-dependent manner, with a more significant impact on root growth compared to shoot growth, and stronger effects observed in grassy weeds than in the broadleaf weed. The integrity of *P. minor* membranes was compromised, as indicated by increased lipid peroxidation, electrolyte leakage, and lipoxygenase activity. Despite an increase in POX activity, the membranes remained vulnerable to damage induced by *β*-pinene. These results suggest that *β*-pinene inhibits root growth primarily by disrupting membrane integrity through oxidative stress. This is in accordance with the mechanism of action of allelochemicals described by Singh et al. [106] and Mutlu et al. [95], where monoterpenoids from EOs can cause oxidative stress in plants and affect growth through the formation of reactive oxygen species (ROS) such as hydrogen peroxide (H_2_O_2_), superoxide radicals (O_2_⁻), hydroxyl radicals (OH^•^), and singlet oxygen (^1^O_2_). ROS can cause disruptions in cellular functions, including lipid peroxidation, protein oxidation, and DNA damage, which ultimately affect plant growth and survival. The excessive production of ROS also activates antioxidant defense mechanisms, which, if overwhelmed, may result in phytotoxic effects such as stunted growth, inhibited seed germination, and reduced root development [108,109,110]. An excess of H_2_O_2_ is generally neutralized by antioxidant enzymes such as catalase (CAT) and peroxidase (POX) [111,112]. Singh et al. [106] found that *α*-pinene increased POX activity in *Cassia occidentalis*. Although CAT activity in ragweed shoots showed no significant change in response to treatments with *Nr*EO or *NcEO*, POX appears to be the major enzyme responsible for the degradation of H_2_O_2_ [37]. In addition, the phytotoxic effects of monoterpenoids could be related to the disruption of cell membranes and the associated enzymes [95,113]. It has been demonstrated that *α*-pinene and limonene can cause phytotoxicity to mitochondrial respiration in the hypocotyls of soybeans (*Glycine max* (L.) Merr). The results suggest that *α*-pinene acts on mitochondria isolated from the coleoptiles and primary roots of maize (*Zea mays* L.) via at least two mechanisms: uncoupling of oxidative phosphorylation and inhibition of electron transfer. Confirming the disruption of mitochondrial energy metabolism, *α*-pinene strongly inhibited mitochondrial ATP production [114,115]. Dmitrović et al. [37] demonstrated that in ragweed shoots, *Nr*EO and *Nc*EO stimulated POX activity, while CAT and superoxide dismutase (SOD) were inhibited, which is consistent with previous findings showing that monoterpenes inhibit SOD [106,116].

Nestorović et al. [35,36] also demonstrated that VOCs released from the shoots of *N. rtanjensis* and *N. sibirica* L. significantly reduced the seed germination and growth of garden cress (*Lepidium sativum* L.) seedlings. The dominant compound in the atmosphere of the *N. rtanjensis* vessel was *trans,cis*-NL, while in the *N. sibirica* vessel, *cis,trans*-NL predominated. The *trans,cis*-isomer showed a significantly stronger inhibitory effect on the seed germination of garden cress than *cis,trans*-NL. The phytotoxic effects of NL are mainly observed through its influence on various biochemical processes that disrupt the plant’s antioxidative system. These perturbations lead to the inhibition of important enzymatic activities, including POX and CAT, which are crucial for the mitigation of oxidative stress. In addition, alterations in the profiles of Fe- and Cu/Zn- isoforms of SOD were detected, further highlighting the altered ability of seeds to neutralize ROS associated with VOCs, in which NL predominates.

To further investigate the phytotoxic effects of VOCs from *Nr*EO, an in vitro system in which seeds were exposed to VOCs in the ambient atmosphere of Petri dishes was established [38]. Species-specific and dose-dependent effects of *Nr*EO were observed. Among the crops tested, *L. sativa* was the most sensitive, while *B. napus* showed the highest tolerance to both the *Nr*EO and the pure monoterpenoids (*α-* and *β-*pinene). Among the weeds, *S. media* was the most sensitive, while *R. crispus* showed the highest tolerance. Of the two monoterpenes, *α*-pinene exhibited greater phytotoxicity than *β*-pinene and particularly affected the seed germination of *L. sativa*, *L. corniculatus*, *B. napus,* and *S. media*. This differs from the results of Nishida et al. [117], who found that *β*-pinene had a stronger inhibitory effect on the seed germination of *Brassica campestris* L. seeds, while *α*-pinene showed no effect on seed germination but later inhibited root growth. In addition, Abrahim et al. [118] found that *α*-pinene inhibited both seed germination and primary root growth in *Zea mays*. Furthermore, Jones et al. [119] attributed the phytotoxic effects of the EOs of *Lamium purpureum* L. and *Lamium amplexicaule* L. to their main constituents *α*-pinene and *β*-pinene. Bozok [101] demonstrated the phytotoxic effect of the EO of *N. nuda* L. subsp. *albiflora*, which contains *trans,cis*-NL as its dominant compound. The major compound of *Nepeta flavida* Hub.–Mor. EO, 1,8-cineole, exhibited phytotoxic effects on seed germination and radicle and plumule length in *L. sativum*, *Raphanus sativus*, and *Eruca sativa* Mill. [100].

The diverse applications of *Nepeta* EOs and NLs, with a specific focus on their stereochemistry, along with the growing global demand for natural and environmentally friendly alternatives to synthetic herbicides, highlight the crucial need for further in-depth research into their mechanisms of action. Further investigations into their phytotoxic effects, modes of action, and interactions with plant and environmental factors are vital for optimizing their effectiveness and broadening their applications.

### 2.3. Advantages of Water Emulsions of Nepeta Essential Oils

**Figure 3:**
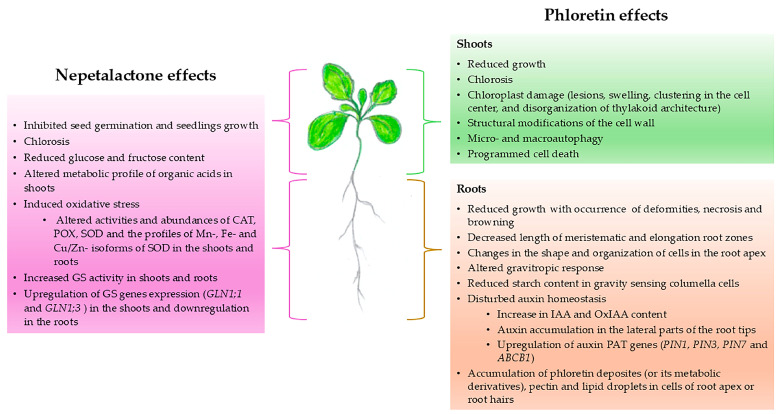
Physiological responses of target plants to nepetalactone or phloretin treatment. CAT—Catalase; GS—Glutamine synthetase; IAA—Indole-3-acetic acid; OxIAA—Oxoindole-3-acetic acid; PAT—Polar auxin transport; POX—Peroxidase; SOD—Superoxide dismutase.

The potential of EOs for weed control in organic farming is promising, but their effectiveness is limited due to rapid evaporation and photolysis. To improve their performance, researchers are developing innovative formulations, such as microencapsulation and the inclusion of natural protectants. These strategies aim to reduce the amount of EO needed and prolong the effect by minimizing volatilization and degradation [120]. The studies discussed in this section demonstrate the potential practical applications of water-based EO emulsions from *Nepeta* species containing volatile monoterpenoid compounds that effectively inhibit the seed germination and growth of weed species. The preparation of a water emulsion with EOs offers many advantages for experimental research and agricultural applications. Since EOs are hydrophobic, their emulsion in water requires the use of stabilizing agents such as emulsifiers in low concentrations. They improve the solubility and uptake of hydrophobic compounds and thus increase their bioavailability. Water emulsions can provide slow or sustained release of EOs. In addition, these formulations require less EO per treatment, making them more cost-effective. Emulsions are easier to apply via spray systems and ensure even distribution across large areas. They also degrade more easily in the environment, supporting eco-friendly practices. Prijović et al. [41] emphasized the potential of *N. rtanjensis* EO as a natural herbicide for the control of invasive and allergenic weeds, with *Stellaria media* (L.) Vill. being the most sensitive. Shekari et al. [121] investigated the inhibitory effect of *N. meyeri* water extracts (*Nm*WEs) from different parts of plants on the seed germination and seedling development of the parasitic plant *Cuscuta campestris* Yunck., which causes significant damage to agricultural crops. *Nm*WEs reduced seed germination and root and shoot growth, as well as the dry weight of *C. campestris* seedlings. Additionally, an inhibitory effect of *Nm*WEs was observed on the activity of key enzymes, such as alpha-amylase, protease, and *β*-1,3-glucanase in germinating *C. campestris* seeds. Dragoeva et al. [122] showed that *Nepeta nuda* L. subsp. *nuda* water extracts (*Nnn*WEs) caused degeneration in *Triticum aestivum* L. and *Cucumis sativus* L. seedling growth, with roots being more affected than shoots. Dmitrović et al. [39] demonstrated that foliar application of *Nr*EO water emulsions triggered an initial stress response in *A. thaliana* that affected metabolism and gene expression. This is consistent with the findings of de Nux et al. [123], who reported that the effectiveness of bioherbicides is limited, as many weed species recover over time. Figure 3 summarises the physiological responses of target plants to treatment with nepetalactone or phloretin as referenced by the authors of this review. The practical use of EO-based formulations requires further research on long-term efficacy, toxicity to non-target organisms, optimal concentrations, environmental impact, synergistic effects, and potential resistance development in weeds.

### 2.4. Exploring the Potential of Nepeta rtanjensis Essential Oil as a Selective Bioherbicide and Crop Protector in Combination with a Broad-Spectrum Synthetic Herbicide—Phosphinothricin

It is well-documented that EOs have gained attention in various fields, especially in the agricultural, environmental, and health sectors, due to their potential benefits as natural alternatives or supplements to synthetic pesticides [4,9,124,125,126]. While EOs can be effective on their own, their combined use with synthetic formulations for crop protection represents an approach to improving sustainability in agriculture. This not only reduces the ecological footprint but also minimizes health risks during handling. By supplementing the synthetic formulations, EOs can result in lower chemical residues in soil, water, and crops, promoting healthier ecosystems and reducing the potential for adverse effects on human and animal health otherwise caused by synthetic herbicides [11,125,126]. While synthetic herbicides often act on a specific group of plants in the field (e.g., dicotyledonous or monocotyledonous and broadleaf or grass weeds), EOs can be more selective and control weeds without harming the crop, which has so far mainly been demonstrated under laboratory conditions. Nestorović et al. [38] found that EO from *Nepeta rtanjensis* (*Nr*EO) and pure monoterpenoids, such as *α*- and *β*-pinene, effectively inhibited the seed germination of *Stellaria media*, while *Brassica napus* showed resistance. In addition, Synowiec et al. [127] showed that crops generally have a greater tolerance to EO than weeds. Of the 12 EOs tested, the most phytotoxic group, consisting of *Carum carvi* L., *Thymus vulgaris* L., *Mentha × piperita* L., and *Salvia officinalis* L. EOs, was rich in oxygenated monoterpenes, while the least phytotoxic EO (*Solidago canadensis* L.) consisted mainly of mono- and sesquiterpene hydrocarbons. Weeds such as *Amaranthus retroflexus* L. and *Centaurea cyanus* L. were the most affected, while corn (*Zea mays* L.) showed the highest tolerance to EO treatments. This result underscores the potential of EOs as selective bioherbicides that provide effective weed control while minimizing negative effects on crops.

Research indicates that the use of herbicide mixtures with different modes of action can be more effective in preventing herbicide resistance in weed species [128,129]. The application of glufosinate in combination with other synthetic herbicides at different crop growth stages has been shown to be an effective strategy for weed control [130,131,132]. This integrated approach improves weed control and reduces the risk of resistance development in weeds by simultaneously targeting multiple biochemical pathways. Nevertheless, antagonistic effects have been reported in some cases [133]. Dassanayake et al. [134] gave an overview of the research on plant-based EOs in combination with insecticides and fungicides and emphasized their potential to reduce the minimum inhibitory concentrations of pesticides to synergistically control plant pests and phytopathogenic fungi. Plant EOs of lavender and thyme can synergize the toxicity of imidacloprid, a conventional insecticide against *Myzus persicae* (Hemiptera: Aphididae), by 16- to 20-fold [135]. Findings from various studies suggest that multiple factors impact the effectiveness of mixed herbicide interactions. Key aspects include the rate of absorption, movement within the plant, metabolic transformation, and competition for common binding sites, among others [129,130,131].

Although some EOs such as lemongrass, pine, clove, cinnamon, and citrus oils have already been commercialized and successfully launched in organic farming [123,136,137], to our knowledge, there is currently no herbicide mixture of synthetic herbicides and EOs on the market. Commercially available organic herbicides are generally intended for weed control in households and small farms, where their effectiveness is limited. In conventional cropping systems, their efficacy is insufficient as many weed species recover over time [123]. A promising transitional solution to overcoming this challenge is the development of formulations that combine synthetic herbicides with bioherbicides. Such formulations are expected to reduce the environmental and health risks associated with synthetic herbicides while improving the effectiveness of weed control and promoting sustainable agricultural practices.

Dmitrović et al. [39] conducted a valuable study that provides deeper insights into the complex interactions between synthetic herbicides and natural bioactive compounds. Contrary to expectations, foliar application of a water emulsion of *Nr*EO rich in *trans,cis*-NL, together with the synthetic herbicide phosphinothricin (PPT)—BASTA^®^ (manufactured by BASF SE, Ludwigshafen, Germany; formerly by Bayer Crop Science AG, Germany), was shown to mitigate phosphinothricin-induced ammonium toxicity in the model plant *Arabidopsis thaliana* (L.) Heynh., grown in a greenhouse, indicating an antagonistic effect. Phosphinothricin inhibits glutamine synthetase (GS), one of the most important enzymes in nitrogen metabolism, which catalyzes the formation of glutamine from glutamate and ammonium ions produced in a variety of metabolic processes and serves as a nitrogen donor for the biosynthesis of all nitrogen-containing organic compounds important for plant growth and development [138,139,140]. The accumulation of toxic levels of ammonium leads to various physiological changes, ammonium toxicity, and lethality [141,142,143,144]. Dmitrović et al. [39] demonstrated that the simultaneous foliar application of *Nr*EO and BASTA alleviated this toxicity by maintaining GS activity, with ammonium concentrations remaining below toxic levels. This protective effect is primarily attributed to NL, the major component of *Nr*EO, although minor components may also contribute to the overall response. Furthermore, Dmitrović et al. [39] highlighted that the cytosolic GS isozymes encoded by the glutamine synthetase 1;1 (*GLN1;1*) and glutamine synthetase 1;3 (*GLN1;3*) genes played a crucial role in *Nr*EO-mediated attenuation of phosphinothricin toxicity. The *GLN1;1* and *GLN1;3* genes were activated shortly after treatment, while *GLN1;3* remained induced even after prolonged exposure. The GS isoforms of *A. thaliana* consist of GLN1;3 subunits, which have lower electrophoretic mobility, and GLN1;1 and GLN1;2 subunits with higher mobility [145]. The application of BASTA and the combined treatment of BASTA and *Nr*EO resulted in the increased activity of certain low-mobility isoforms. This suggests that the BASTA-induced changes in the expression of *GLN1* genes may affect the subunit composition of GS isoforms, which affects their abundance and mobility. Furthermore, the combined application of *Nr*EO with BASTA mitigated the harmful effects of BASTA, resulting in the recovery of fresh weight and glucose and fructose content in *A. thaliana* shoots. In addition, the study of Dmitrović et al. [39] suggests that *Nr*EO could be used as a bioherbicide on BASTA-treated fields to reduce the impact of contamination caused by BASTA residues in the soil on non-target plants. In addition, BASTA may be less effective in fields with medicinal and aromatic plants, such as those with *Nepeta* species, due to the protective effect of their EOs.

Dmitrović et al. [40] further investigated whether the *Nr*EO-mediated reduction of BASTA-induced ammonium toxicity in *A. thaliana*, previously demonstrated during simultaneous foliar application [39], can also be achieved when the herbicide is absorbed via the root. Although BASTA is primarily applied as a foliar spray, its active ingredient PPT has the potential to remain effective in the soil environment. The in vitro system allowed simultaneous exposure of *A. thaliana* roots to BASTA in a sterile culture medium and *A. thaliana* shoots to an atmosphere enriched with VOCs of the *Nr*EO. Simultaneous application of BASTA and *Nr*EO mitigated BASTA-induced phytotoxicity in *A. thaliana* grown in vitro by maintaining physiological and metabolic stability. The combined treatments reduced the negative effects of BASTA on shoot fresh weight and Chl content, indicating the protective role of *Nr*EO in maintaining plant biomass and photosynthetic potential. *Nr*EO modulated the metabolic disturbances caused by BASTA, recovering succinic acid content and sugar accumulation. It also mitigated the inhibitory effects of BASTA on GS activity and normalized nitrogen metabolism, suppressed the excessive accumulation of GS1 proteins, and attenuated the downregulation of *GLN1;3* and *GLN1;2* genes in roots. In addition, *Nr*EO regulated the antioxidant defense system disrupted by BASTA by decreasing the activities of CAT and POX while increasing the activity of SOD in roots, which contributed to enhanced scavenging of ROS. These combined effects emphasize the potential of *Nr*EO to reduce the physiological and biochemical disturbances caused by BASTA and to promote plant resilience under herbicide treatment.

These results suggest that one direction for future research should be to investigate the potential of EOs as biostimulants for crop growth and development. In addition, their role in promoting the production of beneficial bioactive compounds in crops should be investigated, as these compounds are crucial for plant health and stress resistance. In addition, EOs can provide a new approach to improve plant resilience to biotic factors such as pathogens and abiotic stress factors caused by climate change, such as drought, temperature fluctuations, and soil degradation.

## 3. Phytotoxic Effects of Phenolic Compounds

Phenolic compounds are the most studied group of specialized metabolites and bioactive compounds in plants [146,147]. They are involved in the majority of physiological processes in plants, such as plant growth and reproduction, nutrient assimilation, protein synthesis, photosynthesis, fruit quality, enzyme activity, response to biotic and abiotic stress, etc. [146,147]. Phenolic compounds are also some of the most common allelochemicals in ecosystems, with phenolic acids and flavonoids being extensively studied groups with allelopathic effects [148].

### 3.1. Phenolic Acids—Allelochemicals with a Promising Future

Phenolic acids are a diverse class of plant phenolics produced by shikimic acid via the phenylpropanoid pathway [149]. They are usually produced as by-products during the monolignol pathway through the degradation of cell wall polymers such as lignin [149]. In some cases, they can also be produced by microbes [149]. Phenolic acids are divided into cinnamic and hydroxybenzoic acid derivatives [150]. Cinnamic acid compounds have a simple chemical structure of carboxylic acids, consisting of a single benzene ring and a carboxyl group [150]. The most common naturally found and synthetic cinnamic acid derivatives are *p*-coumaric acid, caffeic acid, ferulic acid, chlorogenic acid, sinapic acid, cinnamic acid, quinic acid, dihydrocaffeic acid, dihydrocinnamic acid, 4-fluorocinnamic acid, 4-chlorocinnamic acid, 3,4-dimethoxycinnamic acid, and 4-(3,4-dihydroxyphenyl)-3-buten-2-one. Most synthetic cinnamic acid derivatives are mainly produced through synthetic processes [151] or via the metabolism of specific compounds that plants are exogenously supplied with, such as those containing halogens such as chlorophenylalanines [152]. The group of benzoic acid derivatives includes compounds such as hydroxybenzoic acid, vanillic acid, gallic acid, protocatechuic acid, syringic acid, gentisic acid, benzoic acid, ellagic acid, and salicylic acid [153].

In their comprehensive study, Li et al. [154] highlighted that the presence and positioning of substituents on the benzene ring play a crucial role in determining the phytotoxic potential of phenolic acids. Studies showed that 4-fluorocinnamic acid was the most effective among all cinnamic acid derivatives in terms of the inhibition of seed germination and seedling growth in *Setaria viridis*. Indeed, both halogenated derivatives, 4-fluorocinnamic acid and 4-chlorocinnamic acid, showed significantly higher activity than *p*-coumaric acid, indicating that the halogen atom significantly increased activity compared to the hydroxyl group. Furthermore, the authors concluded that the presence of hydroxyl groups on the benzene ring could reduce phytotoxic activity and that the double bond was not essential for the growth inhibition activity in *S. viridis* seedlings. In addition, the introduction of a methoxy group at meta and para positions to replace the hydroxyl group on the benzene ring of caffeic acid significantly increased phytotoxic activity.

The diverse range of phenolic acids present in plant extracts [155,156,157] complicates the investigation of the allelopathic effects of individual compounds. Thus, investigating plant extracts makes it challenging to pinpoint the specific contributions of individual allelochemicals. To overcome this challenge, scientists often employ standardized pure compounds. However, individual compounds typically achieve similar effects only when applied at concentrations much higher than those found in plant extracts [158,159]. Nonetheless, the overall pattern of allelopathic effects is often consistent, and potential synergistic interactions among these compounds should also be considered.

The inhibition of seed germination and seedling growth are the most investigated effects of phenolic acids [154,156,157,160,161,162,163,164]. These morphological changes are the most visible and easily measurable and also the most useful in agriculture. However, more in-depth studies have shown that exogenously applied phenolic acids also cause physiological changes in plants that damage the ultrastructure of cells and impair their normal function [160,165]. Among the ultrastructural changes, for example, shrinkage and contraction of epidermal cells, together with the formation of larger grooves, channels, and cyst-like structures, were observed in young leaves of *Cassia sophera* L. after 15 days of treatment with water leaf extracts of *Calotropis procera* (Aiton) Dryand. containing nine phenolic acids (caffeic acid, gentisic acid, catechol, gallic acid, syringic acid, ellagic acid, resorcinol, *p*-coumaric acid, and *p*-hydroxy benzoic acid) [165]. Kaur and Kaushik [160] investigated the effect of benzoic acid on mustard (*Brassica juncea* L.). They observed cell damage and irregularities in cell shape and organelle arrangement. In some treatments, dissolution of the middle lamella was observed, while in others, an intact middle lamella with increased wall deposits was present [160]. In addition, chromosomal aberrations and changes in the shape of the interphase and prophase nuclei were observed in the root tips of *Allium cepa* after treatment with leaf extracts of *C. procera* [165], depending on the concentration. The most common aberrations were despiralization in prophase with the formation of micronuclei, sticky anaphase with bridges, sticky telophase, C-metaphase, etc. [165]. Ghost cells, cells with membrane damage, and cells with heterochromatic nuclei were also noticed in treated plants [165].

While phenolic compounds act as natural antioxidants in plants, high exogenous concentrations can stress target plants, triggering oxidative damage. This includes the activation of defense mechanisms and heightened antioxidant enzyme activity and malonaldehyde content due to lipid peroxidation in cell membranes [166]. Politycka et al. [167] demonstrated that exposing cucumber roots to ferulic and *p*-coumaric acids increased the levels of hydrogen peroxide and POX activity.

Phenolic acids could also disrupt the growth of target plants by affecting photosynthetic processes. The results of Patterson [168] indicate that caffeic acid, coumaric acid, ferulic acid, cinnamic acid, and vanillic acid could significantly reduce photosynthetic activity by decreasing Chl content, while Yu et al. [169] demonstrated that benzoic and cinnamic acids reduced leaf transpiration, stomatal conductance, and intercellular CO_2_ concentrations.

Data on the effects of phenolic acids on plant hormones are scarce and often contradictory. For example, benzoic acid derivatives stimulate the degradation of auxins [170], while hydroxybenzoic acid promotes the degradation of both auxins and gibberellins [171]. Garg and Garg [172] reported that caffeine and chlorogenic acids significantly increased the activity of indole-3-acetic acid (IAA) oxidase in chickpea roots 10 days after treatment. On the other hand, treatment of wheat seedlings with ferulic acid resulted in increased concentrations of IAA, gibberellins, cytokinins, and abscisic acid (ABA), which led to the inhibition of seedling growth [173]. When applied at high concentrations, 3,4-dihydroxybenzoic acid inhibited the growth of tobacco callus and roots, as well as the regeneration of shoots, while at lower concentrations, it stimulated callus formation and rooting processes, acting similarly to auxins [174].

The allelopathic role of phenolic compounds in ecosystems has been recognized since ancient times, with phenolic acids often being the dominant components in most plant extracts, defining their allelopathic potential [175]. Despite the well-documented phytotoxic effects, bioherbicides containing phenolic acids have not been commercialized, unlike those based on EOs or their components [29]. Further in-depth studies on their effects, particularly their mechanisms of action, are crucial for paving the way toward their commercialization. As noted, the complexity of plant extract compositions undoubtedly poses a significant challenge in this regard.

### 3.2. Flavonoids—A Diverse Group of Compounds with Phytotoxic Activity

Flavonoids account for more than 10,000 known compounds, many of them with allelopathic potential. They are synthesized via the phenylpropanoid or acetate-malonate pathway [176]. All flavonoids share the basic C6-C3-C6 structural skeleton, which consists of two aromatic C6 rings (A and B) connected by a three-carbon unit usually organized as a heterocyclic ring (C) containing an oxygen atom [146]. They are typically categorized into several subgroups based on the carbon of the C ring on which the B ring is attached and the degree of unsaturation and oxidation of the C ring [177]. Flavonoids in which the B ring is attached at position 3 of the C ring are referred to as isoflavones, while those with the B ring linked at position 4 are classified as neoflavonoids. Flavonoids with the B ring attached at position 2 can be further categorized into several subgroups, depending on the specific structural characteristics of the C ring. Flavanones and flavanols are characterized by a single bond between the second and third carbon atoms of the C ring, whereas flavones, flavonols, isoflavones, and anthocyanidins feature a double or aromatic bond at this position. Chalcones are characterized by A and B rings linked by an open C ring (three-carbon bridge). In plants, flavonoids occur as aglycones, glycosides, and methylated derivatives [177,178]. Glycosides can be either O-linked or C-linked. The variant of flavonoid glycoside is based on the number of positions on the flavonoid for glycosylation, the degree of glycosylation, and the number of sugars involved in glycosylation [146].

Flavonoids have various physiological functions in plants, such as regulating seed germination and seedling growth, attracting pollinators, influencing flower and fruit coloration, and defense against abiotic stress. Flavonoid accumulation has been observed in response to abiotic factors such as heat, cold, UV radiation, and nutrient deficiencies. Additionally, flavonoids play protective roles through detoxification, antimicrobial activity, and phytoalexin production [179]. Flavonoids are known as antioxidants due to their characteristic low redox potential. They can therefore reduce strong free radicals such as superoxides, alkyl radicals, and hydroxyl radicals [180]. The position of the B ring, along with the number and placement of hydroxyl groups on the catechol group of the B ring, influence the antioxidant activity of flavonoids [181]. The functional hydroxyl groups of flavonoids can donate electrons via resonance to stabilize free radicals and provide antioxidant protection [182].

Flavonoids are mostly found in the vacuole in glycosylated form [168]. They are excreted from the surface of leaves and other aerial parts of the plants [183] and roots [176], so they can act directly in the rhizosphere to affect other plants. In general, the main phytotoxic effects of flavonoids are inhibition of seed germination and seedling growth, inhibition of auxin transport and energy metabolism, including blocking mitochondrial and chloroplast functions, hyperpolarization of cell membranes, and influencing enzyme activity [180]. Examples of the phytotoxicity of flavonoids are numerous and include a large number of plant species, of which we will mention only a few. One of the good examples of the effects of flavonoids is plants that exhibit both autotoxic and allelopathic effects, such as alfalfa (*Medicago sativa* L.) [176]. Other crops such as wheat (*Triticum aestivum* L.), tomato (*Lycopersicon esculentum* Mill.) [184], barley (*Hordeum sativum distichum*) [180], radish (*Raphanus sativus* L.), and garden cress (*Lepidium sativum* L.) [185] were also evaluated. The flavonoids had different negative effects on the seed germination and radical elongation of these plants. Garden cress was more sensitive to the treatments than radish [185]. Detailed studies on the influence of flavonoids on seed germination and growth included the model plant *Arabidopsis thaliana* (L.) Heynh., as well as a large number of weed species such as spotted knapweed (*Centaurea maculosa* Lam.), diffuse knapweed (*Centaurea diffusa* Lam.), Dalmatian toadflax (*Linaria dalmatica* L.), and kochia (*Kochia scoparia* L.) [184]. In addition, catechins secreted by the invasive plant *C. maculosa* inhibit the seed germination and growth of *C. diffusa* and *A. thaliana* [180].

Not all flavonoids are necessarily inhibitory for the treated plants. Similarly to phenolic acids, it is also known that the application of flavonoids in small amounts can have a stimulatory effect, while higher concentrations have an inhibitory effect, a phenomenon known as hormesis [185]. In the case of garden cress, flavone and 3-OCH_3_-flavone in particular inhibited radical elongation, while quercetin, *β*-naphthoflavone, catechin, catechol, and the flavanones naringin and hesperetin promoted radical elongation in the same species [185]. Most flavonoids had an inhibitory effect on root growth, including flavones (7-OCH_3_ flavones, chrysin dimethyl ether, and 3′,4′-di-OCH_3_ flavones), morin, and 7-OCH_3_ flavanones [185].

These tests are useful for comparing phytotoxic effects with the flavonoid structure. Thus, flavones were found to have the strongest inhibitory effect on seed germination and seedling growth because they contain the 4-oxo functional group in their structure, as well as a 2,3-double bond in the C-ring and one or more hydroxyl groups [185]. Flavonols, which have a similar structure to the flavones and contain a hydroxyl group in a C-3 position, also show a strong inhibitory effect. Furthermore, the presence of a 4-carbonyl functional group combined with a C-2–C-3 double bond is crucial for sustaining their effects. It is assumed that the most active compounds are the flavonoids, of which the methylated ones are the most potent. Conversely, biological activity is not affected by glycosylation or by the absence of a 3-OH group in ring C and a 5-OH group in ring A. De Martino et al. [185] hypothesize that the catechol orientation in the B-ring could be responsible for the biological effects.

One of the most important mechanisms of action is the influence of flavonoids on the regulation of auxin transport and degradation [176]. Depending on their structure, flavonoids can influence auxin degradation through IAA oxidases and peroxidases [176]. In addition, they affect the polar transport of auxin and thus impair the root growth of the treated species. Certain flavonoids have been reported to stimulate the destruction of IAA by IAA oxidase due to specific structural requirements. These compounds can also inhibit or interfere with the mode of action of plant hormones such as auxin [186].

Processes related to auxin transport and energy metabolism in plants are interconnected and a number of physiological changes occur under the action of flavonoids, including disruption of cellular respiration and chloroplast function, hyperpolarization of cell membranes, alteration of the pH of intercellular spaces, impairment of enzyme activity, etc. [176,187].

Some flavonoids are potent inhibitors of energy metabolism and block the functions of mitochondria and chloroplasts. For example, flavones have been shown to impair ATP formation in plant mitochondria. Flavonoids have an affinity for many enzymes and other proteins in plants, including those required for mitochondrial respiration, where they lead to the inhibition of nicotinamide adenine dinucleotide hydrogen (NADH) oxidase and disruption of the balance of reactive oxygen species [177]. In addition, flavonoids take on a negative charge in the intracellular medium at a neutral pH. At low concentrations, these compounds can promote cell growth, perhaps due to more effective utilization of cellular enzymes, proteins, and electron carriers. High concentrations of flavonoids, on the other hand, may act as membrane hyperpolarizers and alter the ATP pump, making the flavonoids toxic to cells and thus reducing their growth [188].

### 3.3. Phytotoxic Effects of Chalcones and Their Potential as Future Bioherbicides

**Figure 4:**
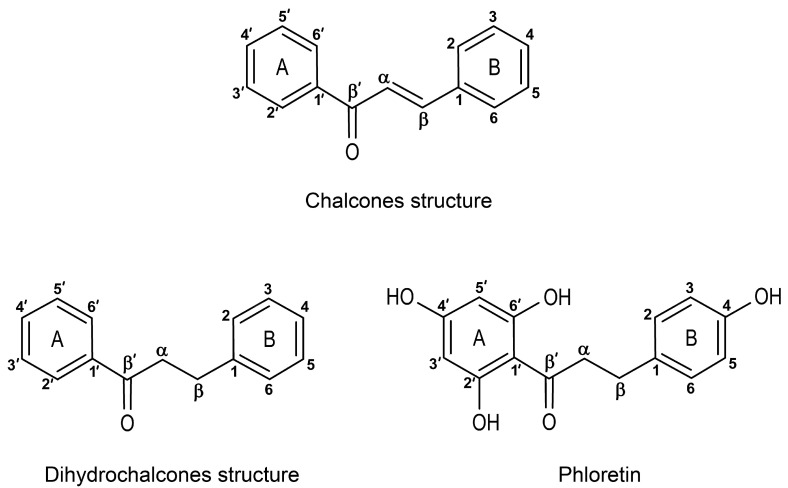
The chemical structures of chalcones, dihydrochalcones, and phloretin.

Chalcones are a class of natural compounds belonging to the flavonoid family characterized by an open-chain structure containing two aromatic rings (A and B) linked by a three-carbon *α,β*-unsaturated carbonyl system (Figure 4). This unique structural framework imparts chalcones with a high degree of chemical reactivity and multifunctionality, making them key intermediates in the biosynthesis of various flavonoids and isoflavonoids in plants [189]. About 250 chalcone aglycones and O-glycosides have been reported as naturally occurring components in 54 plant families, predominantly in dicotyledonous plants but also in some monocotyledonous plants, pteridophytes, and gymnosperms [190]. Chalcone glycosides are usually found as yellow flower pigments in the genera *Dahlia, Coreopsis,* and *Cosmos* [191,192], and in species such as *Paeonia trollioides* Stapf ex Stern, *Dianthus caryophyllus* L, *Aeschynanthus partifolius* R.Br., and *Asystasia gangetica* (L.) T.Anderson [193]. Chalcone-containing plants (Liquorice (*Glycyrrhiza glabra* L)., Kava-kava (*Piper methysticum* G.Forst.), *Boesenbergia rotunda* (L.) Mansf., and *Lophira alata* (Van Tiegh. Ex Keay) have been used as traditional remedies for a range of medical issues [194,195,196,197].

The phytotoxic potential of chalcones attracted the interest of scientists two decades ago, starting with research on *trans-*chalcone (1,3-diphenyl-2-propen-1-one). Chen et al. [198,199] demonstrated that *trans*-chalcone intensively inhibits the growth of shoots and roots in 20 annual plant species. Soon after, Yun et al. [200] suggested that one of the key enzymes in the biosynthesis of lignin monomers, 4-coumarate: coenzyme A ligase (4CL), is a potential target for chalcone-induced inhibition of plant growth. The results of Chen et al. [201] indicated that growth suppression in soybean (*Glycine max* (L.) Merr.) cells by exogenous chalcone is positively associated with the inhibition of lignin biosynthesis. Negative effects of chalcones on the root growth of *A. thaliana* seedlings were demonstrated by Díaz-Tielas et al. [202], who reported that *trans-*chalcone at a concentration of 12.5 μM inhibited root growth by 30% compared to the control, while a concentration of 1200 μM led to a striking inhibition rate of 94%. Furthermore, a marked reduction in root hair density was also observed, becoming apparent at 25 µM and pronounced at 50 µM of *trans*-chalcone. This compound also disrupted starch reserves in amyloplasts, impairing the normal gravitropic response in recipient plants. Apical meristematic cells of *A. thaliana* roots treated with 35 µM chalcone (IC_50_) exhibited characteristic markers of programmed cell death, including mitochondrial condensation, organelle disintegration, and chromatin fragmentation. Programmed cell death was further confirmed by acridine orange/ethidium bromide staining, which corroborated the loss of mitochondrial transmembrane potential observed following chalcone exposure [202].

*trans*-Chalcone exhibited phytotoxic effects on various plant species, inhibiting seed germination in *Plantago lanceolate* L. and *Lactuca sativa* L. and early root growth in *Amaranthus retroflexus* L., *Echinochloa crus-galli* L., and *P. lanceolate* [203]. In *A. thaliana*, *trans*-chalcone impaired both development and adult plant physiology, with its effects varying depending on whether it was applied via watering or spraying [203]. Treatment with *trans-*chalcone also disrupted the aerial part of the plants. For example, *A. thaliana*, *A. retroflexus,* and *Triticum aestivum* L. plants exhibited pronounced chlorosis of leaves during the treatment with *trans*-chalcone [202,203]. The progressive chlorosis of *A. thaliana* shoots induced by *trans*-chalcone is linked to early plasma membrane depolarization and significant disruptions in chloroplast structure and function. These changes are accompanied by ROS accumulation, pigment degradation, impaired photosynthesis, and programmed cell death [204].

Transcriptomic analysis revealed that many of the affected genes were transcription factors and genes associated with oxidative stress, xenobiotic detoxification, ABA and auxin biosynthesis, and primary metabolic processes [205]. Kyoto encyclopedia of genes and genomes (KEGG) pathway analysis of transcriptome results indicated that pathways associated with stress (e.g., specialized metabolism, glutathione metabolism, and hormone signal transduction) were mostly affected, as well as amino acid metabolism and sulfur and phenylpropanoid metabolism pathways. Proteasome function, endoplasmic reticulum protein processing, and peroxisome function genes were also significantly affected [205].

Research shows that many chalcones exhibit potent herbicidal effects with minimal toxicity to crops. Their efficacy depends on the substituents on the A and B rings, the applied concentrations, and the specificity of the target species or tissues. Chotsaeng et al. [206] synthesized 45 different chalcone derivates, among which chalcone 14f, with a thiophenyl group on ring A and a phenoxyacetic acid group on ring B, was the most potent compound against seed germination and growth in Chinese *amaranth (Amaranthus tricolor* L.) and barnyardgrass (*Echinochloa crus-galli* L.). At the highest concentration (1600 µM), this chalcone completely inhibited the seed germination of Chinese amaranth and highly reduced seed germination (42%) and shoot (51%) and root length (79%) in barnyardgrass. Perera et al. [207] reported several naturally occurring chalcone derivates, isolated from the extract of *Ambrosia salsola,* with moderate phytotoxic effects. Salsolol A and B showed phytotoxcity against duckweed (*Lemna paucicostata* Hegelm.), while the other two structurally similar dihydrochalcones had no activity. The chalcone derivatives, 2′,3′,4′,3,4-pentahydroxychalcone and 2′,3′,4′-trihydroxychalcone are potent inhibitors of phosphoenolpyruvate carboxylase (PEPC), a critical enzyme for carbon fixation and biomass production in the C_4_ photosynthetic pathway of many of the world’s most problematic weeds, for example, pigweed (*Amaranthus* species), foxtail (*Setaria* species), johnsongrass (*Sorghum halepense*), dallisgrass (*Paspalum dilatatum*), crabgrass (*Digitaria* species), barnyardgrass (*Echinochloa* species), and velvetleaf (*Abutilon theophrasti*) [208]. At the whole-plant level, both chalcone derivatives, 2′,3′,4′,3,4-pentahydroxychalcone and 2′,3′,4′-trihydroxychalcone, significantly inhibited the growth of the C4 weed *A. retroflexus* without affecting oilseed rape, a C3 plant [208].

Chalcones and their synthetic derivatives show considerable potential for developing bioherbicidal compounds as alternatives to commercial herbicides, particularly due to their minimal toxicity to crops. Their effectiveness in controlling weed species in small-scale households and organic farming could be significant depending on their structure, dosage, and method of application. However, further investigations of their mechanisms of action, persistence in soil, and impact on non-target plants, the soil microbiome, insects, and other animals are vital for optimizing their efficacy and integration into sustainable weed control practices.

### 3.4. Dihydrochalcones—The Most Abundant Apple Phenolic Compounds

Dihydrochalcones (DHCs) represent a distinct group of chalcones characterized by two aromatic rings linked by a saturated three-carbon bridge, where the double bond between *α* and *β* C atoms, which is typical of chalcones, is reduced (Figure 4). All DHCs are glycosylated derivatives of phloretin aglycon, differing in their sugar groups and/or binding sites. Due to its typical DHC structure, and a lack of a carbohydrate ligand, phloretin (Figure 4) is a highly flexible molecule capable of efficiently interacting with biological macromolecules, possessing a broad spectrum of biological activities [209,210,211,212,213].

A specific natural source of DHCs is the genus *Malus*, including the domesticated apple (*Malus × domestica* Borkh.), where DHCs account for 96–97% of the total phenolic content in leaves [214,215]. Phlorizin (phloretin 2′-O-glucoside) is the earliest identified and most abundant DHC in the genus *Malus,* making up 4–18% of the dry mass of apple leaves [216]. Substantial amounts of phlorizin are also found in the roots, stems, and bark of apple trees, from which it was first isolated in 1835 [217]. The key step in phlorizin biosynthesis is the glycosylation of the aglycone phloretin (3-(4-hydroxyphenyl)-1-(2,4,6-trihydroxyphenyl)-1-propanone) at the 2′ position, catalyzed by a specific uridine diphosphate-glycosyltransferase that directly determines the concentration of phlorizin in apple tissues [218]. Due to its high prevalence, phlorizin serves as a key parameter for classifying *Malus* species, determining phylogenetic and phytogeographic relationships within the subfamily Maloideae [219], and as a marker in the food industry for detecting the presence of apple in fruit juices [220].

Advancements in analytical techniques have enabled the detection of phlorizin in trace amounts in other species within the Rosaceae family, such as strawberry (*Fragaria × ananassa* Duch.) [221] and dog rose (*Rosa canina* L.) [222], as well as species from unrelated families such as Fabaceae and Ericaceae [223,224,225].

#### 3.4.1. Can Dihydrochalcones Be Considered Allelochemicals?

This question was first raised by Börner in 1959 [226], who attributed the presence of dihydrochalcones to the development of Apple Replant Disease (ARD). This disease manifests as stunted growth of both tree and root systems, alongside poor fruiting performance in seedlings planted on sites of previous apple orchards [227]. Examination of the root systems of ARD-affected trees has revealed the degradation of epidermal cells and cortical tissues, as well as a significant reduction in the development of lateral roots [228,229]. Additionally, a pronounced reduction in the number of functional root hairs has been noted [230]. Although the challenge of establishing apple orchards on soil previously used for apple cultivation has been documented for over two centuries, the etiology of ARD remains highly controversial. The involvement of phenolic compounds in the development of ARD remains a significant question, given the numerous but still controversial pieces of evidence regarding the influence of rhizosphere microorganisms and nematodes [231].

Although Börner hypothesized that phlorizin and phloretin, detected in apple orchard soils, might play a role in ARD symptom development [226,232], their auto-allelopathic activity remained largely unexplored for decades. However, Zhang et al. [233] and Bai et al. [234] demonstrated that root exudates from wild apple species *Malus pumila* and *Malus prunifolia* inhibit the seed germination and seedling development of their own species. In addition, the results of Yin et al. [235] imply that dihydrochalcones released into the soil via root exudation or decomposition of plant residues (fallen leaves, fruits, bark, or roots) could be the key factors in ARD symptom development. Nicola et al. [236] observed that treating Fuji apple seedlings with a mixture of soil and shredded roots from the M26 rootstock variety resulted in a significant reduction in Chl content, root mass, and total plant biomass after four months. Ultra-high-performance liquid chromatography (UHPLC) mass spectrometry analysis revealed a substantial increase in the levels of phlorizin and phloretin in the treated mixture compared to the control substrate. Jianghong et al. [237] suggested that low concentrations of phlorizin (1.0 μM) stimulated growth in *Malus hupehensis* Rehd. seedlings, while higher concentrations (1–4 mM) exerted inhibitory effects, causing ultrastructural changes in the chloroplasts and mitochondria of leaf and/or root cells. High concentrations of phlorizin also elevated malondialdehyde (MDA) content, an indicator of membrane lipid peroxidation, as well as the activity of the antioxidant enzymes SOD, POX, and CAT. Furthermore, elevated phlorizin concentrations were associated with reduced transpiration and photosynthesis rates [237]. Similar effects of phlorizin were observed in seedlings of the wild apple species *M. micromalus*, where increased activities of SOD, POX, and CAT, as well as elevated levels of MDA and proline, were recorded in proportion to the applied concentration and treatment duration [238]. Wang et al. [239] reported a reduction in respiration rates and the activity of enzymes involved in the tricarboxylic acid cycle in roots of *M. hupehensis* treated with 4.0 mM phlorizin. Additionally, phloretin and phlorizin, implemented at concentrations detected in orchard soils, increased the expression levels of proteins associated with defense mechanisms in root cells of *M. hupehensis,* as reported by Yin et al. [240]. Treatments with these compounds upregulated genes related to defense system-related and free radical-scavenging proteins in the roots, indicating that phlorizin and phloretin could be involved in the onset of ARD. Recently, Xiang et al. [241] analyzed the correlation of *Fusarium* and *Mortierella* species and phlorizin content in the soil and soil organic matter with ARD severity. Structural equation modeling indicated that the degree of occurrence of ARD was affected by microorganisms, phlorizin, and organic matter in the soil. All these findings suggest that dihydrochalcones, either exuded from roots or released during the decomposition of apple organic matter, could play a role in the development of ARD symptoms, either directly or indirectly, by affecting rhizosphere microorganisms.

#### 3.4.2. Phytotoxic Potential of Dihydrochalcones

Apple dihydrochalcones that enter the soil through root exudation or the decomposition of plant material may exhibit inhibitory effects on other plant species, including weeds. A significant challenge in studying such allelopathic interactions in the rhizosphere lies in the complexity of isolating and identifying root-exuded compounds, as well as the intricate nature of rhizosphere ecosystems and the activity of microorganisms that can modulate the effects of allelochemicals. To eliminate factors affecting the quality and quantity of exuded allelochemicals, a model system of an in vitro culture of genetically transformed apple roots was recently established in several apple cultivars [31]. UHPLC mass spectrometry confirmed that genetic transformation did not alter the levels of major specialized metabolites in apple roots such as phlorizin, glycosylated and aglycosylated flavonoids, and phenolic acids compared to non-transformed apple roots. The established in vitro culture system of transformed apple roots thus provides a controlled environment for studying the apple’s allelopathic potential in the absence of microorganisms and other disruptive factors from the rhizosphere [31].

The phytotoxic effects of apple root exudates were evaluated using the model plant *A. thaliana.* The findings revealed that apple root exudates did not inhibit seed germination but exerted significant phytotoxic effects on seedling growth and development, reducing root and shoot growth by 35.1 and 62.1%, respectively [31]. Notably, the exudates had the most pronounced impact on lateral root formation, decreasing the number of lateral roots by 74% after 10 days of treatment. The authors demonstrated that root growth inhibition in seedlings is associated with disrupted expression of cyclin-dependent kinase (CDK) genes [31], which play a central role in cell cycle regulation and the evolutionarily conserved mechanism of cell division in eukaryotes [242]. CDKs belong to a family of serine/threonine protein kinases, whose substrate specificity is determined by binding to the corresponding cyclin (CYC) and whose activity is regulated by a series of CDK activators and inhibitors. Treatment of *A. thaliana* seedlings with apple root exudates for 10 days reduced the expression levels of cyclin-dependent kinases *CDKA1;1* and *CDKB2;1* and cyclins *CYCA3;1* and *CYCB2;4* in shoots, while gene transcript levels in roots were unaffected [31]. The analysis of specialized metabolites in the apple root exudates identified the presence of dihydrochalcones, including phlorizin (8.01 ng mL⁻^1^; 0.017 µM) and phloretin (7.49 ng mL⁻^1^; 0.027 µM), alongside caffeic acid (14.58 ng mL⁻^1^; 0.08 µM) and chlorogenic acid (8.82 ng mL⁻^1^; 0.025 µM).

Based on these findings and the existing literature data on phloretin’s role in ARD, as well as its known multifunctionality and reactivity, further research investigated its phytotoxic effects on species beyond the *Malus* genus. [32,33,34].

#### 3.4.3. Phloretin as a Plant Growth Inhibitor

Smailagić et al. [32] demonstrated that phloretin acts as a potent growth inhibitor that causes severe morphological abnormalities and agravitropic responses in *A. thaliana* seedlings. The authors investigated phloretin’s effects in concentrations ranging from 125 to 1500 µM and observed significant dose- and time-dependent inhibition of seedling growth. Root development and primary root elongation were more strongly inhibited than leaf growth, with lateral root formation significantly suppressed, especially at concentrations above 500 µM. High phloretin concentrations (750–1500 µM) initially stimulated adventitious root formation, likely as a stress response, but prolonged exposure stunted their growth and induced root necrosis and browning [32]. In contrast to *A. thaliana*, where root growth inhibition gradually increased over time and peaked at the end of treatment (86.6% at 1500 µM after 15 days of treatment), the inhibition in *C. bursa-pastoris* seedlings was highest at the start (59.5% on day 5 with 1000 µM) but remained stable or even decreased in the subsequent days [34]. Phloretin similarly suppressed lateral root development in *C. bursa-pastoris*, which was particularly evident at concentrations of 500 and 1000 µM (41.5 and 41.6%, respectively, after 10 days of treatment). However, the inhibitory effect was less pronounced compared to *A. thaliana,* where reductions of 75.6 and 58.9% were observed at the same concentrations. The disparity in phloretin’s phytotoxic effects between these two related species was further evident in the inhibition of shoot growth. In *C. bursa-pastoris*, shoot growth inhibition progressively increased over the treatment period, peaking at 29.7% in 14-day-old seedlings grown on media with 1000 µM phloretin. Under identical conditions, *A. thaliana* seedlings exhibited nearly double the inhibitory effect, with a 48.1% reduction in shoot growth after 14 days at the same phloretin concentration.

Phloretin treatment also disrupted the gravitropic response of *A. thaliana* seedlings, leading to serious disturbances in root architecture and histology [32]. The frequency and type of deformities were dose- and time-dependent, with 500 µM phloretin inducing the most abnormalities. A significant reduction in the starch content of columella cells was also observed, disrupting gravitropic perception in treated seedlings. Histological analysis revealed disorganized columella cells, shortened meristematic and elongation zones, and hypertrophied epidermal and cortical cells detaching from underlying layers [32].

Using histochemical staining, Đorđić et al. [34] detected an increased presence of phenolic compounds, lipids, and pectin in the roots of *C. bursa-pastoris* seedlings treated with 500 µM phloretin. The blue coloration observed in root cells following Toluidine Blue O staining indicated the accumulation of phloretin and/or its metabolic derivatives. As phloretin readily interacts with various biological molecules, plants tend to quickly metabolize phloretin into more stable, water-soluble glycosylated forms that can then be actively transported into vacuoles. The excess accumulation of lipids observed in the axial root tips and root hairs of treated seedlings suggests disrupted lipid degradation or inefficient energy utilization due to growth stagnation [34]. The authors highlighted that the accumulation of pectin in the root border cells presumably serves as a mechanism to inhibit phloretin uptake by binding to and reducing its concentration within the cells. It is well-established that covalent bonding, as well as multiple hydrogen bonds and hydrophobic interactions, can occur between polysaccharides, such as pectin, and polyphenols [243,244,245]. Fernandes et al. [246] demonstrated direct interactions between phlorizin and pectic polysaccharides, specifically arabinans from sugar beet. They proposed that hydrophobic regions formed by polymer entanglement trap the phlorizin molecules in a manner similar to dextrins [247].

#### 3.4.4. Phloretin Modulates Auxin Homeostasis in Roots

IAA, as the primary biologically active form of auxin, plays a central role in plant development and in coordinating plant responses to fluctuating environmental conditions by regulating processes such as cell division, elongation, and differentiation. The key mechanism underlying auxin function is based on finely coordinated processes of biosynthesis, conjugation, degradation, and polar auxin transport (PAT), which together establish local maxima and morphogenetic gradients within plant tissues. Smailagić et al. [32] reported that excessive production and accumulation of IAA and oxindole-3-acetic acid (OxIAA) in the lateral root regions plays a key role in the morphological abnormalities and agravitropic response observed in phloretin-treated roots. The hormonal metabolic profile revealed a rapid increase in IAA levels shortly after the application of 500 µM phloretin (2.2- and 3.2-fold at 2 and 6 h of treatment, respectively), accompanied by a decrease in auxin biosynthetic precursors. These results indicated that seedlings strived to reduce auxin levels and maintain auxin homeostasis in roots by degrading excess IAA to OxIAA through the rapid conversion of IAA-Glu and IAA-Asp. Using the *A. thaliana* transgenic line expressing the auxin-responsive reporter DR5rev::GFP, the authors demonstrated an intensive accumulation of auxin in the lateral root cap, as well as in the epidermis and cortical cells, which are key components of the main basipetal (shoot-ward) auxin transport pathways in roots [32].

PAT plays a central role in auxin maxima generation, which involves the active influx and efflux of auxin through specific groups of transporters, including AUXIN1/LIKE AUXIN1 (AUX1/LAX), PIN-FORMED (PIN) auxin efflux proteins (PIN1, PIN2, PIN3, PIN4, and PIN7), and proteins from the MULTI-DRUG RESISTANCE/P-GLYCOPROTEIN (MDR/PGP) subfamily, which belongs to the ATP-BINDING CASSETTE (ABC) transporter superfamily (ABCB1, ABCB4, ABCB19) [248,249]. The normal gravitropic response in roots depends on regular auxin flow in roots mediated by symmetrically positioned PIN3. Auxin reaches the basipetal stream via PIN2, while ABCB1 and ABCB4 transporters facilitate its shoot-ward transport from the root apex [250,251,252]. Gene expression analysis [32] revealed that phloretin significantly upregulates *PIN1, PIN3, PIN7*, and *ABCB1* genes in roots, contributing to agravitropic growth and morphological abnormalities in *A. thaliana* seedlings.

#### 3.4.5. Phloretin’s Effects on Mesophyll Cell Ultrastructure and Antioxidative Status

Phloretin’s effects on leaves, such as chlorosis and leaf decay, could be associated with ultrastructural damage to the mesophyll cells in phloretin-treated *A. thaliana* seedlings. Smailagić et al. [33] demonstrated that short-term phloretin treatment (10 days) with 250 and 500 μM phloretin induced swelling in the chloroplast, its ultrastructural disorganization, and reduced starch synthesis, all indicating an impaired photosynthetic process. Prolonged exposure (28 days) triggered micro- and macroautophagic responses and pro-apoptotic changes, underscoring its substantial impact on cellular integrity. The authors also reported an association between ultrastructural changes in leaf mesophyll cells and a significant reduction in Chl a and Chl b content following both short-term and prolonged treatment with 500 µM phloretin. A reduced Chl/carotenoid ratio in treated seedlings reflected cellular stress and observed ultrastructural changes [33]. A similar decrease in Chl content was also observed in *C. bursa-pastoris* seedlings treated with 500 µM phloretin [34].

Allelochemicals present in the rhizosphere can provoke oxidative stress in plants. The balance between the generation and removal of ROS plays a vital role in regulating plant growth, development, and stress adaptation. Phytotoxic allelochemicals that disrupt photosynthetic processes often cause an accumulation of ROS. As a result, assessing the antioxidant capacity of target plants is a critical step in determining the effectiveness of bioherbicidal agents. Smailagić et al. [33] demonstrated that treatment with 500 µM phloretin induced a significant increase in the antioxidant capacity (TAC) and total phenolic content (TPC) of *A. thaliana* seedlings while reducing malondialdehyde levels and SOD, CAT, and POX activities. The authors suggest that the notably high TPC and TAC observed in phloretin-treated seedlings are likely due to the absorption of phloretin from the nutrient medium. Given phloretin’s known antioxidant potential, this emphasizes the importance of the hydroxyl group at the 2′-position of the dihydrochalcone A ring structure for its free radical-scavenging activity. These findings highlight that phloretin induces cellular stress without exacerbating oxidative damage.

#### 3.4.6. The Prospects of Phloretin as a Bioherbicidal Agent

Phloretin’s ability to target multiple physiological processes in plants, coupled with its various health-beneficial properties, suggests its potential for use in sustainable weed control practices. Moreover, through the development of phloretin derivatives with enhanced herbicidal properties, it may be possible to create novel, eco-friendly bioherbicides capable of managing herbicide-resistant weed species. However, future research is essential for the development of bioherbicides based on phloretin. To ensure that these bioherbicides are both effective and safe for widespread use, further studies are needed to fully understand the mechanisms through which phloretin exerts its phytotoxic effects on weeds. This includes investigating its mechanism of action, optimal application methods, and potential effects on non-target organisms and ecosystems. Additionally, research should focus on improving the stability and bioavailability of phloretin, as well as exploring its potential for synergistic effects when combined with other natural compounds. With continued research and innovation, phloretin-based bioherbicides could offer a sustainable, eco-friendly solution for weed management, providing an alternative to synthetic herbicides and contributing to the reduction of herbicide resistance.

## 4. Limitations of Using Plant Products as Bioherbicides

Despite the numerous benefits that bioherbicides offer, there are certain limitations that need to be considered [124,253]. One of the main challenges is their relatively short half-life, which, while beneficial for minimizing environmental toxicity, may compromise their availability and efficacy over time. In addition, bioherbicides often require certain environmental conditions to work optimally, such as sufficient light, appropriate temperatures, humidity, soil moisture levels, etc. Their effectiveness can also be affected by factors such as the timing of application and soil type [254]. In some cases, bioherbicides work more slowly than chemical herbicides, so longer times are required to achieve the desired effects. In addition, plants from the same region or within the same taxonomic group do not produce equal amounts of specialized metabolites. It is, therefore, important to select representative genotypes based on their composition and the quantity of bioactive compounds [255]. A deeper understanding of how environmental factors and their interactions with genotypic components influence the aromatic profile of *Nepeta* species provides a strategic way to predict and optimize the production of valuable metabolites. Moghaddam et al. [254] demonstrated that the composition of EO in *N. binaludensis* varies depending on ecological conditions. They found that the amount of NL was negatively correlated with altitude. Additionally, populations of *N. binaludensis* growing in high, cold, rainy, and steep areas had lower levels of NL and higher levels of 1,8-cineole, and vice versa. In addition, some specialized metabolites such as alkaloids and cyanogenic glycosides can be toxic to humans and animals [124], necessitating detailed studies on their effects on non-target organisms. As noted by De Mastro et al. [256], allelochemicals affect processes such as photosynthesis and respiration, but are not species-specific, making them unsuitable as selective bioherbicides. One of the biggest challenges in the development of bioherbicides is the great diversity of weed species, which requires customized solutions. Regulatory approval can be lengthy and costly, limiting adoption, while scaling up production and ensuring product stability remain significant obstacles [257].

### 4.1. Solution Stability and Soil Biodegradability of Nepetalactone and Phloretin

The long-term stability of specialized metabolites with practical applications, such as bioherbicides, is of crucial importance. The stability of NL in soil has not yet been tested and will be the focus of our future research. Lockhart et al. [258], however, indicate the stability of *cis,trans*-NL, *trans,cis*-NL, nepetalic acid (NA), and dihydronepetalactone (DHNL) in dry plant samples (using ethanol as a solvent and calculated as % of compound per dry weight of plant samples, *w*:*w*), in dry extracts after the evaporation of ethanol (calculated as % of compound per dry weight of extract, *w*:*w*), and in extracts dissolved in ethanol (calculated as mg of compound per mL of ethanol, *w*:*v*) of *N. cataria* stored under ambient light or in darkness. Using UHPLC-QTOF/MS or UHPLC-QQQ/MS, the concentrations of the compounds were analyzed over two years to determine the best storage methods, although a one-year study is required for pharmaceutical products according to the Food and Drug Administration (FDA) [259]. These results are important for possible commercialization and for establishing storage conditions that help maintain the efficacy of bioherbicides whose active ingredient is NL. *Cis,trans*-NL in dry plant samples of *N. cataria* was degraded faster compared to *trans,cis*-NL. When exposed to light, the total amount of NL decreased by 98% after two years. When the samples were protected from light, the total NL content was ten times higher than in the light-exposed group. In both treatments, the content of *cis,trans*-NL decreased to almost zero after only two months. *Trans,cis*-NL in the extract was degraded more slowly than *cis,trans*-NL. After 16 months, the samples stored in darkness contained 6.76% *trans,cis*-NL, which was six times higher than in the samples exposed to light. During long-term storage of extracts with a high *trans,cis*-NL content, light protection significantly increases stability. In samples with ethanol solution, *cis,trans*-NL degraded faster than *trans,cis*-NL, although the degradation was slower than in the extracts. In both light-exposed samples and samples stored in darkness, the amount of *cis,trans*-NL reached zero after 16 months. The trend of constant *trans,cis*-NL concentrations occurred in the samples with ethanol solution, and the concentration remained at approximately 7 mg mL^−1^ throughout the 24-month experiment in darkness. The DNL content remained stable over the two years, whereas the NA content increased from 1.046 to 1.926% in the extract samples stored in the darkness and to 1.744% in the light-exposed samples. NA concentrations were increased in both groups, which could be due to the hydrolysis of NL [258]. A high water content has been shown to significantly accelerate the degradation of various bioactive compounds in extracts, solutions, or emulsions of EOs, while ethanol solutions of extracts have been shown to be more effective in minimizing the degradation of iridoids [258]. In addition, crude plant extracts often require encapsulation or other advanced formulations to preserve their stability.

Currently, there is a lack of sufficient data regarding the persistence of dihydrochalcones in the soil, which limits our understanding of their behavior and stability in soil environments. Pedrinho et al. [260] provided the first evidence of the low soil persistence of dihydrochalcone, isoflavone, and aliphatic phenol. The dissipation of dihydrochalcone was between 1.20 to 10.23 days in three different soil types (Dijon, France; Prüfbericht, Germany; and commercially available soil from LUFA Speyer, Speyer, Germany) [260]. The obtained results also indicated a slower dissipation of these three plant-derived compounds in the Prüfbericht soil compared to the other two soils. No dose-dependent pattern in the dissipation of dihydrochalcone, isoflavone, and aliphatic phenol was evident, with the exception of the Prüfbericht soil, where dihydrochalcone and isoflavone exhibited substantially higher DT_50_ (time for concentration to decrease to 50% of its initial value), and DT_90_ (time for concentration to decrease to 10%) values at the higher dose rate.

Rapid degradation of pinocembrin dihydrochalcone was also observed, with a DT_50_ ranging from 0.85 to 1.7 days and a DT_90_ between 4.55 and 5.26 days [261]. The study of the adsorption of pinocembrin dihydrochalcone showed the character of a Freundlich curve model, indicating that pinocembrin dihydrochalcone interacts with the soil via functional groups on soil constituents that vary in quantity and quality and influence the strength of adsorption. The measured partition coefficient (KF) of 0.0661 indicates a low affinity to soil, which means that pinocembrin dihydrochalcone is probably not strongly bound to soil particles. This result suggests that pinocembrin dihydrochalcone may leach out of the soil more rapidly, reducing its potential for long-term soil persistence and efficacy.

Given that the phytotoxicity of phloretin or its derivates has not been tested in greenhouse or field conditions, there are no available data in the literature on its stability in soil. However, the half-life of phloretin in soil is likely influenced by various factors, including microbial activity, soil pH, composition, weather conditions, and other environmental variables. Zhang et al. [262] reported that phloretin is stable in acidic conditions and high temperatures but prone to degradation in alkaline and strongly oxidizing conditions. Several studies have examined the chemical composition and levels of phloretin and phlorizin in the soil of apple orchards affected by ARD and changes due to environmental and positioning factors [235,263,264]. Jiang et al. [265] reported that the W6 strain of *Ochrobactrum haematophilum*, commonly found in soil and the plant rhizosphere, was capable of degrading dihydrochalcones in replanted soil. The W6 strain exhibited significant activity in degrading phlorizin, phloretin, ferulic acid, cinnamic acid, and *p*-hydroxybenzoic acid in replanted soil by 30.00, 32.26, 48.10, 42.86, and 70.00%, respectively, compared to control soil.

Considering the relatively short half-life of the dihydrochalcones studied in soil [260,261], it is crucial to conduct a detailed stability analysis of phloretin. This analysis is necessary to better understand its persistence and behavior in the soil environment before proceeding with field experiments, ensuring more reliable and accurate results in practical applications. These results are essential to evaluate their long-term effects and effectiveness, particularly in agricultural and environmental contexts.

### 4.2. Enhancement of Bioactivity Using Nanotechnology

To address limitations such as the high volatility, instability, and rapid degradation of specialized metabolites in EOs and the extracts, solutions, and water emulsions of EOs under environmental conditions, Mondéjar-López et al. [266] highlight the benefits of using polysaccharides as raw materials for encapsulating natural chemicals in nanoparticles as novel crop protection products. Polysaccharides can be derived from various natural sources, including plants (hyaluronic acid, pectin, cellulose, lignin, *β*-glucan, starch, and guar gum), animals (chitosan and chitin), algae (agar, alginate, and carrageenan), and microorganisms (pullulan, xanthan gum, dextran, and bacterial cellulose). These materials offer many advantages, including biocompatibility, biodegradability, and the controlled release of active compounds. As the authors point out, various natural substances, such as EOs, plant extracts, antimicrobial compounds, and microRNA, can be incorporated into these nanoparticles and delivered to target sites in plants, easily passing through cell walls and membranes due to their small size range of 10 to 1000 nm. In addition, bioactive compounds can mediate hormone signaling and gene expression in various physiological aspects of plants and increase their tolerance to abiotic stress conditions. In addition, this type of treatment can support sustainable agriculture by properly developing food safety through the production of non-toxic nanoparticles, low-cost industrial scalability, and the use of biodegradable materials. To date, no results have been reported on the encapsulation and use of nanoparticles for iridoid compounds in *Nepeta* species for potential bioherbicide applications. However, Amighi et al. [267] encapsulated EOs from *N. hormozganica* and *N. dschuprensis* into chitosan using a simple ionic gelation method with tripolyphosphate. The synthesized nanocapsules were evaluated for antifungal activity and demonstrated that encapsulated EOs increased antifungal efficiency by up to 100%. Cheraghipour et al. [268] showed that the encapsulation of *N. cataria* EO in a chitosan nanocomposite had a lethal effect against *Toxoplasma gondii*. Mahalleh et al. [269] investigated the encapsulation of *N. binaludensis* extracts and their release kinetics under laboratory conditions. Extracts were encapsulated using maltodextrin and gum arabic and the release kinetics of the microencapsulated phenolic compounds were studied. The results revealed that combining maltodextrins with dextrose and gum arabic yielded superior efficiency in assessing antioxidant activity. Zhang et al. [270] prepared microcapsules containing EOs from *Nepeta* sp. species through electro-atomization and complex coacervation, using sodium alginate and gelatin as wall materials. The results indicated that the microcapsules enabled stable release over 15 days and maintained a mosquito repellency rate above 80% for 48 h, suggesting potential applications in mosquito repellent formulations. Hogenbom et al. [271] demonstrated that cyclodextrin inclusion complexes successfully encapsulated and enhanced the properties of *N. cataria* EO, extending its potential use in tick control formulations. Sari et al. [272] investigated the in vivo activity of biosynthesized silver nanoparticles with *N. cataria* extract in a rat excision wound model, where the treatment significantly improved wound healing. Mani et al. [273] presented an eco-friendly approach for managing disease-transmitting vectors and bacterial pathogens by rapidly synthesizing copper nanoparticles using *N. cataria* leaves. Encapsulation and nanoparticle formulations of EOs and extracts from *Nepeta* species have demonstrated enhanced stability and improved biological and pharmacological responses in various studies. These advancements underline their potential and will be a focus of future research, particularly in evaluating their efficacy as bioherbicides under agricultural conditions.

To date, the encapsulation of phloretin for bioherbicidal applications has not been explored. Nevertheless, several studies have examined different encapsulation techniques to enhance the solubility, chemical stability, and bioavailability of phloretin for pharmaceutical and medical applications. Phloretin is known for its human health-beneficial properties. Its beneficial effects are primarily linked to its strong antioxidant properties. Additionally, it influences multiple signaling pathways and molecular mechanisms, providing therapeutic advantages for a range of diseases, such as cancer, diabetes, liver and kidney injuries, encephalomyelitis, ulcerative colitis, asthma, arthritis, and cognitive impairments [274]. The authors discussed the pharmacological actions, underlying mechanisms, and molecular targets of phloretin. Moreover, the review provides insights into physicochemical and pharmacokinetic characteristics and approaches to promote the pharmaceutical development of phloretin for its therapeutic applications in the future, as well as scientific efforts to enhance its bioavailability by modifying its physicochemical properties and molecular structure.

Phloretin is known to dissolve readily in methanol, ethanol, acetone, dimethyl sulphoxide, and alkaline solutions. However, its low water solubility and low stability make its usage in clinical applications difficult, especially in water-based formulations. To enhance its dissolution, lipid-based delivery systems, such as nanoemulsions (NEs), solid lipid nanoparticles (SLNs), and nanostructured lipid carriers (NLCs), offer a promising approach. These systems are advantageous because the lipids used are biocompatible and biodegradable. NLCs, a sophisticated delivery system made from a combination of solid and liquid lipids, forms an imperfect matrix that efficiently incorporates and protects bioactive compounds [275]. NLCs offer several benefits, including high encapsulation efficiency, stability, non-toxicity, suitability for industrial processing, and sustained release. Gu et al. [276] prepared a phloretin-loaded NLC using high-pressure homogenization. Transmission electron microscopy revealed that the NLC had a spherical shape with uniform distribution, an average particle size of 137.40 ± 3.27 nm, and a PdI value of 0.237 ± 0.005. The encapsulation efficiency was 96.68 ± 0.06%. In vitro release studies demonstrated that the phloretin NLC effectively sustained phloretin release compared to the phloretin ethanol solution.

Several studies have reported the successful encapsulation of phloretin and/or phlorizin using cyclodextrins. Cyclodextrins have a wide range of applications in food, pharmaceutical, medical, cosmetic, and environmental applications due to their low cost, safety, biocompatibility, and biodegradability [277]. Ishizuka et al. [278] thoroughly investigated the inclusion complexes of cyclodextrins with phlorizin, while Aree et al. [279] demonstrated that encapsulation with hydroxypropyl-β-cyclodextrin enhances water solubility and preserves the antioxidant capacity of entrapped phlorizin. The encapsulation of phloretin and phlorizin in cyclodextrins leads to perpendicular conformations of their pharmaceutically active forms, further highlighting the potential of cyclodextrins as molecular stabilizers and aqueous solubilizers for improving the bioavailability and efficient delivery of bioactive compounds in food [279]. Hu et al. [280] demonstrated the improved water solubility of phloretin after encapsulation with methyl-β-cyclodextrin and 2-hydroxy propyl-β-cyclodextrin using the freeze-drying method. The reducing power and 1,1-diphenyl-2-picryl-hydrazyl (DPPH) radical-scavenging activity tests revealed that the antioxidant activity of phloretin improved after the formation of inclusion complexes. Sharma et al. [281] designed polymer-based nanoparticles as effective oral drug delivery systems capable of enhancing both the systemic bioavailability and therapeutic efficacy of phloretin. Phloretin-loaded polymeric nanocomposites were prepared using ionic gelation and optimized for yield, encapsulation, loading, particle size, PdI, and zeta potential. Results indicated enhanced oral bioavailability and antioxidant capacity, as well as reduced myocardial infarct size in an ischemia-induced rat model by ~46% versus ~13% for free phloretin, showing significant cardiomyocyte protection and ROS suppression.

To facilitate the transdermal administration of phloretin, polymeric microspheres based on gallic acid were obtained by a reverse-phase emulsion radical polymerization reaction and characterized by transformed infrared spectroscopy [282]. The tests revealed that particle swelling increases over time, enhancing the release of phloretin from the matrix. Preliminary studies investigating the release behavior of phloretin demonstrated that the particles efficiently release phloretin upon contact with the skin, showing performance comparable to that of a free drug solution. Furthermore, the microspheres successfully protected phloretin from degradation, as confirmed by mass spectrometry analysis. Antioxidant activity testing in rat liver microsomes revealed time-dependent effects, with higher activity observed in microspheres loaded with phloretin compared to unloaded ones. Collectively, these findings suggest that gallic acid-based microspheres have potential as transdermal carriers for phloretin, offering protection and therapeutic benefits against UV radiation-induced skin damage.

Mariadoss et al. [283] reported potent antioxidant and anti-carcinogenic effects of phloretin-loaded chitosan nanoparticles in 7,12-dimethylbenz[a]anthracene (DMBA)-induced oral cancer in experimental animals. Over the past two decades, chitosan-formulated nanoparticles have been extensively investigated for their efficiency in delivering chemotherapeutic drugs to target cancer cells. Numerous studies have highlighted the superior biocompatibility and biodegradability of phytochemical-encapsulated chitosan nanoparticles compared to other nanoparticle formulations. Treatment with phloretin-loaded chitosan nanoparticles significantly reduced tumor volume and neoplastic changes and enhanced mitochondrial-mediated apoptosis in tumor cells.

Chen et al. [284] chemically modified a phytoglycogen structure to introduce negative surface charges via carboxymethylation (CMPG) and then prepared CMPG-based ternary nanocomplex particles. This was achieved via electrostatic interactions with sodium caseinate (forming the core) and chemical cross-linking with pectin (forming the shell). The resulting nanocomplex demonstrated remarkable efficiency in encapsulating phloretin, a model lipophilic bioactive compound. Moreover, it exhibited slow and sustained release of phloretin under simulated gastrointestinal conditions and significantly enhanced its antioxidant activity in an aqueous environment compared to pure phloretin dissolved in ethanol.

The collected studies suggest that encapsulating phloretin is an effective strategy for improving its solubility, chemical stability, and bioavailability, making it suitable for pharmaceutical and medical applications. Furthermore, these encapsulation techniques hold great potential for developing phloretin-based bioherbicidal formulations, supporting organic and sustainable agricultural practices.

### 4.3. Effect of Nepetalactone and Phloretin on Non-Target Organisms

#### 4.3.1. Effects on Pollinators and Non-Pollinating Insects

Detailed studies on how specialized metabolites, such as nepetalactone and phloretin, affect non-target organisms when used as bioherbicides are essential. It is well-known that many *Nepeta* species exhibit repellent activity [81,285,286], and it is crucial to carefully assess the potential effects of prospective bioherbicides on beneficial pollinators. While data on the effects of NL on non-target organisms are limited, studies on EOs from other species in the *Lamiaceae* family provide valuable insights. The toxicity of EOs from mint, oregano, and thyme was tested on two pollinators, *Apis mellifera* and *Trigona hyalinata*. These EOs did not act as repellents, but they reduced the time pollinators spent in treated areas with sublethal doses. Oregano and thyme EOs decreased the survival of *A. mellifera*, while mint EO was less toxic and more selective. Despite the potential of EOs in weed control, their use must be carefully assessed due to possible negative impacts on pollinators [287].

Despite evidence that phloretin and its derivatives are present in significant quantities in apple floral pollen [288] and in *Camellia oleifera* bee honey [289], data on the effects of phloretin on insect pollinators remain limited. Honeybees (*Apis mellifera*), mason bees (*Osmia* spp.), bumblebees (*Bombus* spp.), and hoverflies are considered the primary pollinators of apple flowers, transferring pollen grains from the anthers to the stigmas [290]. Although there are no studies on the effects of phloretin on these pollinators, research by Sharma et al. [291] showing that honeybees prefer foraging on apple floral pollen suggests that the phloretin and its glucosides present in the pollen are not harmful to them. However, further studies on a broader range of phloretin concentrations are necessary to assess the dose-dependent effects on pollinators.

Considering the impact of phloretin on non-pollinating insects, studies report conflicting findings regarding phloretin and its derivatives, identifying them as either repellents or attractants for various insect pests of apple trees. For example, phlorizin present in apple leaves repels aphids such as *Myzus persicae* and *Amphorophora agathonica* [292], yet it attracts *Rhopalosiphum insertum* and the green apple aphid (*Aphis pomi*) [293]. Interestingly, larvae of *Spodoptera littoralis* have been observed to avoid feeding on previously damaged apple leaves, a behavior associated with increased levels of phlorizin detected in the leaves several days after injury [294]. Furthermore, the percentage of defoliation and the extent of damage on apple leaves caused by adult Japanese beetles (*Popillia japonica*) were found to be inversely proportional to the phlorizin concentrations in the leaves of 10 *Malus* species. Additionally, the inclusion of phlorizin and phloretin in artificial diets significantly reduced the feeding intensity of Japanese beetles [295]. Patton et al. [296] demonstrated that natural compounds prevalent in the rose family (Rosaceae) exhibited dose-dependent effects on the feeding behavior of adult Japanese beetles, with phloretin inhibiting feeding at concentrations between 1 mM and 10 mM. Finally, the populations of soil mites and Collembolans were significantly reduced in soils affected by ARD [297].

Plant β-glucosides such as phloretin can negatively impact herbivorous insects by inhibiting insect trehalose activity. Trehalose is the primary circulating sugar in insects, serving as both an energy reserve for flight and a cryoprotectant. Trehalose concentration in the hemolymph is crucial for regulating carbohydrate intake and maintaining nutritional homeostasis [298]. Given its significance, any disruption in trehalose activity can adversely affect insect metabolism, leading to significant developmental and locomotor problems or death [299,300,301]. The effects of phlorizin and other plant β-glucosides, such as amygdalin, prunasin, mandelonitrile, and esculin, were assessed for their ability to reduce tissue trehalose activity in insects (*Tenebrio molitor*, *Musca domestica*, *Spodoptera frugiperda*, and *Diatraea saccharalis*). Among the tested β-glucosides, phlorizin showed the strongest inhibitory effect on trehalose activity in the Malpighian tubules and midgut cell membrane fractions of *Diatraea saccharalis*, while exhibiting mild or negligible effects on other insects [302].

Mitchell et al. [303] found that phloretin and other plant flavonoids such as flavone, chrysin, apigenin, kaempferol, morin, quercetin, and myricetin also dose-dependently inhibit cytochrome P-450-dependent ecdysone 20-monooxygenase activity in adult female *Aedes aegypti*, the wandering stage of *Drosophila melanogaster,* and in the fat body and midgut of last instar larvae of *Manduca sexta*. Ecdysone 20-monooxygenase is the enzyme system that hydroxylates ecdysone at C-20 of the side chain to form ecdysterone, which is involved in the regulation of insect development and metamorphosis [304]. It is involved in an increasing number of physiological processes in insects, including the biosynthesis of endogenous pheromones and hormones [305], the detoxification of natural and synthetic xenobiotics in insects [306], and the biosynthesis of long-chain hydrocarbons [307].

In light of the previously outlined effects of phloretin, it is vital to expand research on its impact, especially on bees and other pollinators, to better understand its potential risks. Such studies are necessary to determine the safety of phloretin for use in agricultural settings and field applications, ensuring it does not pose any harm to pollinator populations or the broader ecosystem.

#### 4.3.2. Effects on Soil Microbiota

Bioherbicides, in addition to being effective in controlling the germination and growth of weed species, should not have negative effects on soil microorganisms. The soil microbiome is a complex community of microorganisms, including bacteria, fungi, archaea, viruses, and protozoa. These microorganisms play a vital role in organic matter decomposition, nutrient cycling, nitrogen fixation, and the establishment of symbiotic relationships with plants, all of which directly influence soil fertility and plant health [308]. To date, the effects of NL, a potential bioherbicide from *Nepeta* sp., as well as EOs, water emulsions, and extracts from this plant, on the biogeochemical properties of soil and key microbial groups have not been studied. This will be a critical focus of future research to enhance our understanding of *Nepeta’*s potential in sustainable agriculture and ecosystem conservation. However, few studies have investigated the impact of EOs from the *Lamiaceae* family on soil microorganisms. While *Nepeta* sp. EOs and extracts are widely recognized as antimicrobial agents [55,309], some microorganisms can be stimulated by their presence and utilize them as a source of carbon dioxide and energy. Vokou and Liotiri [310] discovered that EOs extracted from *Origanum vulgare*, *Rosmarinus officinalis* L., *Mentha spicata* L., and *Coridothymus capitatus* (L.) Rchb.f enhanced microbial respiration. Similarly, EOs from *Lavandula stoechas* L. increased microbial respiration due to bacterial growth stimulation [311]. In contrast, Khare et al. [312] reported a decrease in microbial biomass and activity following treatment with emulsions of *Ocimum basilicum* and *Mentha arvensis* L. EOs. Jouini et al. [313] demonstrated that EOs from *Thymbra capitata* L. at concentrations of 8 and 12 µL mL^−1^, when added to soil, and *Mentha × piperita* EO at a concentration of 20 µL mL^−1^ have herbicidal potential against *Avena fatua*, *Echinochloa crus-galli*, *Portulaca oleracea*, and *Amaranthus retroflexus*. The same authors also investigated the effects of different concentrations and exposure durations of EOs on the biochemical properties of the soil and the main microbial groups. The addition of *T. capitata* EO stimulated soil microorganisms, with the effects varying depending on the applied dose and the duration of the incubation period. An increase in microbial biomass carbon and nitrogen, as well as respiration, along with a reduction in extractable carbon, occurred immediately after the addition of the lowest EO dose (4 µL mL^−1^). This suggests that the carbon substrates available in the soil, together with those added by the EO treatment, were simultaneously immobilized and mineralized by the soil microorganisms. In contrast, as the EO dose increased, the stimulation initially affected only microbial respiration. Towards the end of the incubation, the stimulatory effects of the two lowest doses of *T. capitata* (8 and 12 µL mL^−1^) disappeared, while the highest oil dose (12 µL mL^−1^) led to a reduction in microbial biomass carbon and microbial activity. This suggests that the highest dose of *T. capitata* EO destroyed some of the soil microorganisms, and the remaining microorganisms were unable to utilize cytoplasmic materials released from the cells, as confirmed by the increased extractable carbon and low respiration rate compared to the control. The results indicate that *T. capitata* EO at the highest concentration can be harmful to soil microorganisms, and these negative effects become apparent after approximately two months. Both bacteria and fungi were slightly reduced upon the addition of *M. × piperita* EO compared to the control at all doses of the oil (12, 16, and 20 µL mL^−1^). However, by day 50, all microbial groups did not show significant deviations from the control. These studies, which present conflicting results, indicate that if EOs, extracts, and water emulsions are to be used in the field for integrated weed management, further research is needed to clarify their impact on soil microorganisms. Therefore, optimizing doses and treatment methods is essential to minimize harmful effects while maintaining weed control efficiency. Sustainable strategies for using bioherbicides are key to ensuring long-term soil health.

Pedrinho et al. [260] conducted q-PCR analysis to determine the potential effect of the dihydrochalcone applied at an agronomic dose rate of (×1; 0.6 μg/g soil) and (×10; 6 μg/g soil) on the abundance of broad taxonomic groups, such as total bacteria and fungi, in three different soil types (Dijon, France; Prüfbericht, Germany; and commercially available soil from LUFA Speyer, Germany). The authors demonstrated that dihydrochalcone induced a significant dose-dependent decrease in the abundance of total bacteria in the Dijon soil 28 days after application, an effect that was not replicated in the other two types of soil. In contrast, fungal populations were unaffected in all of the tested soils. The authors further extended their analysis to ammonia-oxidizing microorganisms (AOMs), which are important for the nitrogen cycle. AOMs include ammonia-oxidizing archaea (AOA; *Nitrososphaerales* and *Nitrosotaleales*) and ammonia-oxidizing bacteria (AOB; *Nitrosospira, Nitrosomonas,* and *Nitrosococcus)*. They concluded that dihydrochalcone significantly changed the percentage difference in AOA (−58.9 to 208.5%) and AOB (−48.5 to 57.2%) depending on the soil type.

Considering that phloretin and phlorizin are the main components of apple exudates [31] and were linked to ARD in previous studies [236,264], data on the influence of ARD-affected soil could be valuable in evaluating the impact of phloretin on soil microbiota. ARD-affected soil causes changes in the soil microbiome due to complex interactions between living organisms, external factors, and soil properties [297,314]. It is evident that ARD alters the microflora to the detriment of beneficial organisms, resulting in the proliferation of microorganisms that impair apple tree growth, such as the oomycetes *Pythium* and *Phytophthora*, the fungi *Cylindrocarpon* and *Rhizoctonia*, Actinomycetes, and bacteria such as *Bacillus* and *Pseudomonas* [315,316,317].

Genetic sequencing of ARD soil samples showed that eukaryotes accounted for only 1.96% (±0.2) of the total reads, the majority of which were classified as fungi (58.7% ± 2.27), while archaea and viruses accounted for less than 0.55 and 0.07% of the total reads, respectively. Bacteria from the phyla Proteobacteria, Actinobacteria, Bacteroidetes, and Acidobacteria were the most abundant in the rhizosphere metagenomes [318]. In ARD-affected soils, the genera *Friedmanniella*, *Terrabacter*, and *Thermoleophilum* were the most reduced compared to uncontaminated soils. Additionally, a reduced number of reads were found for the genera *Mycobacterium*, *Pedosphaera*, and *Solirubrobacter*. In contrast, the genera *Opitutus*, *Dongia*, and *Novosphingobium* were more frequently represented in the soil samples affected by ARD. The genomic sequencing results of ARD soil samples in Italy indicated that ARD soils were dominated by the phyla Proteobacteria, Actinobacteria, Acidobacteria, and Bacteroidetes, which accounted for 24 and 71% of all sequences, respectively [319].

In conclusion, there is a potential for phloretin to influence changes in the soil microbiome, which may have significant implications for soil health and plant growth. However, further research is necessary to fully understand the direct effects of phloretin applied at different concentrations. These studies should focus on the impact of phloretin on soil microbial communities, their functional roles, and the broader ecological implications. Such investigations are crucial to determine the feasibility and effectiveness of phloretin as a tool in sustainable agricultural practices.

#### 4.3.3. Effects on Crops

Bioherbicides possess the potential for phytotoxic effects on crops, necessitating precise and judicious application to ensure crop safety and effective weed management. While data on the effects of phloretin on crops are not yet available and studies are still in the experimental phase, some insights into the impact of nepetalactone have already been documented. Nestorović Živković et al. [38] conducted in vitro tests to evaluate the effects of *N. rtanjensis* EO on the germination of weed and crop seeds. Among the weed species, *Rumex crispus* seeds showed a high level of resistance to the *N. rtanjensis* EO, while the lowest concentrations of the EO completely inhibited the germination of *Stellaria media* seeds. Regarding crops, concentrations as low as 0.3% of the EO significantly reduced the germination of *Lepidium sativum* seeds, while concentrations of 0.5% completely inhibited the germination of *Lactuca sativa* seeds. However, the *N. rtanjensis* EO, even at the highest concentration, had no effect on the germination of *Brassica napus* seeds. When using specialized metabolites as bioherbicides, careful selection of species and doses is crucial for effective weed control while minimizing crop damage. Laboratory studies offer initial guidance, but field conditions, including environmental variability and biotic interactions, can impact efficacy. Further field research is needed to determine optimal doses and application methods that ensure crop safety and sustainable weed management.

### 4.4. Regulatory and Economic Challenges Associated with the Application of Nepetalactone and Phloretin in Commercial Agriculture

The application of bioherbicides in commercial agriculture offers promising alternatives to synthetic chemicals, but their production and economic feasibility require the careful consideration of various factors. The chemical synthesis of compounds such as NL can be challenging and expensive, especially due to the presence of different stereoisomers of NL. Producing a specific isomer requires precisely controlled reaction conditions, which can increase synthesis costs. Furthermore, the process may involve multi-step reactions, each carrying additional costs, including the use of specialized reagents, catalysts, and equipment [83,320]. On the other hand, using a natural source, such as the distillation of NL from plants, may be a more economical option. Extraction from plants typically involves simpler processes, such as distillation or solvent extraction, which are less demanding in terms of equipment and reagents. This approach also has the advantage of lower costs compared to chemical synthesis. However, production from natural sources may be limited by the availability of raw materials and seasonal variations in plant yields, which can increase costs [321].

The use of phloretin for bioherbicidal formulations largely involves its isolation from natural sources, primarily domesticated apples. Phloretin could be obtained from apple trees residues (leaves, bark, and roots) through the deglycosylation of phlorizin, which is present in enormously large quantities in apple tissues (from 4 to 18% of leaf tissue DW, depending on the cultivar and the season) [216]. In view of the relatively expensive and time-consuming extraction of phloretin, the need arose to find more economical solutions for the rapid production of large quantities of phloretin. Recently, Liu et al. [322] constructed an artificial biosynthetic pathway for phlorizin through a modular engineering strategy. They enabled the production of phloretic acid, phloretin, and phlorizin in *Escherichia coli*. Producing phloretin or its derivatives in microbial cell factories could provide a fast and cost-effective approach for their use in sustainable weed management practices.

Comparing the effect of NL with other known phenolic compounds such as ferulic acid or juglone, the following can be observed. Treatment with a ferulic acid water solution at a concentration of 10^−2^ M (1.94 mg mL^−1^) results in 95% and 98% inhibition of the seed germination of *Rumex crispus* and *Amaranthus retroflexus*, respectively [323]. NL (2 and 4%) in the atmosphere of Petri dishes had no effect on the germination of *R. crispus* [41], while 0.05 and 0.1% NL in a water emulsion caused 95 and 100% inhibition of *A. retroflexus*, respectively [38,41]. Juglone at a concentration of 10^−3^ M (0.174 mg mL^−1^) caused 100% inhibition of germination of *Lepidium sativum* [324], while 4% NL in the atmosphere of Petri dishes caused 65% inhibition [38]. It would be valuable to investigate the potential synergistic effect of volatile NL and soluble phloretin to potentially reduce the required amount of bioactive compounds, making use of these substances more efficiently and cost-effectively. Based on our previous research, *A. thaliana* would be the next target plant we would investigate as we have demonstrated the inhibitory effect of both NL and phloretin on it. In our future work, we also plan to investigate the combined effect of NL and phloretin on other weed species, as well as on non-target organisms.

However, the scientific results summarized in this review represent just the first step to introducing nepetalactone and phloretin as potential bioherbicides. Registering a natural product, such as refined EO from *N. cataria* [325] or phloretin, as a biopesticide requires navigating through intricate regulatory frameworks. These frameworks include a detailed characterization of the active ingredient, focusing on its physicochemical properties, followed by a thorough assessment of human health risks. Key evaluations include toxicity, the potential for eye and skin irritation, genotoxicity, inhalation risks, dose–response analysis, and the possibility of contaminating drinking water. Additionally, a complete cumulative effect assessment is required. Equally important is the ecological evaluation, which involves studying the environmental behavior of the product, the risk of groundwater contamination, and potential impacts on non-target organisms, including endangered and protected species. Given the complexity of these requirements, further research is essential to refine nepetalactone and phloretin in bioherbicide formulations and application methods. Such research will ensure that bioherbicides can be effectively integrated into sustainable agricultural systems while minimizing risks to human health, the environment, and crop productivity.

## 5. Conclusions

Plants produce and release diverse allelochemicals to suppress the seed germination and growth of neighboring plants, thereby securing vital resources such as space, water, nutrients, and light. A variety of compounds from EOs are volatile and exert their effects through the air, while the others, including phenolic compounds, are secreted into the soil via root exudates or released by the decomposition of plant residues. Once released in the air or soil, these compounds affect the growth, vitality, and reproduction of surrounding plants. Plant allelochemicals usually act synergistically, maximizing their impact when combined. This further complicates their identification and the study of their mechanisms of action. Nonetheless, knowledge of the allelopathic activity of these compounds has been constantly growing. Identifying new phytotoxic compounds is crucial for exploring novel mechanisms and sites of action to combat herbicide-resistant weeds. The recently discovered phytotoxic effects of nepetalactones and phloretin have the potential to significantly advance sustainable agricultural practices. Their multifunctionality, particularly their diverse modes of action, offers significant potential for the development of novel bioherbicides that could serve as effective tools against herbicide-resistant weeds. Moreover, mixtures of allelopathic extracts or their active components with known synthetic herbicides could represent a promising strategy to reduce the dependence on synthetic herbicides in crop protection. By enhancing the effectiveness of synthetic herbicides at lower concentrations, these combinations could minimize environmental impact, reduce toxicity, and improve the sustainability of agricultural practices. This area of research could lead to the development of more environmentally friendly alternatives that support the transition from weed control strategies using synthetic herbicides to sustainable crop protection.

## Figures and Tables

**Table 1:** Documented phytotoxic activity of *Nepeta* essential oils.

*Nepeta* Species	Dominant Compounds of the EO	Species Examined for Sensitivity to EO	Experimental Design	Reported Phytotoxic Activity	Ref.
*N. binaludensis* Jamzad *N. elymaitica* Bornm *N. menthoides* Boiss & Buhse	1,8-Cineole	*Avena ludoviciana* Dur. *Sinapis arvensis* L.	In vitro assay in Petri dishes on filter paper. Dose tested: 1, 2, 4, and 8 μg EO mL^−1^ water with Tween 20 (0.1 g 100 mL^−1^ of water). In a greenhouse assay, foliar application: 1.25, 2.5, 5, and 10% EO in water (*v*/*v*) on 3-week-old weeds.	Inhibition of seed germination and root and shoot length by increasing the concentration of EOs after 2 weeks of treatment. Inhibition of *S. arvensis* seed germination (100%) at *N. binaludensis* EO 4 μg mL^−1^ and 8 μg mL^−1^, and at *N. menthoides* EO at 8 μg mL^−1^. Reduction of Chl content and electrolyte leakage and increase in proline levels after 7 days of treatmnets. *S. arvensis* was more sensitive than *A. ludoviciana*.	[97]
*N. mahanensis* Jamzad & Simmonds	Nepetalactone				
*N. cataria* L.	*trans,cis*-Nepetalactone (55.0%) *cis,trans*-Nepetalactone (31.2%)	*Avena fatua* L. *Hordeum spontaneum* Koch *Lepidium sativum* L. *Nepeta cataria* L. *Ocimum basilicum* L. *Taraxacum officinale* (L.) Weber ex F.H.Wigg.	In vitro assay in Petri dishes on filter paper. Dose tested: 150, 300, 600, and 1200 µL EO L-1 of water.	Inhibition of seed germination (100%) at 1200 µL L-1 in all species tested except *H. spontaneum*, where it was 26%. Inhibition of seed germination (100%) at 600 µL L-1 in *A. fatua* and *T. officinale*. Inhibition of seedling length and fresh and dry weight with increasing EO concentration.	[94]
*N. cataria* L.	*cis,trans*-Nepetalactone (77%) Spathulenol (4.26%) Caryophyllene oxide (4.80%)	*Ambrosia artemisiifolia* L.	In vitro enclosed volatile bioassays—shoot growth and activities and abundance of antioxidant enzymes. Dose tested: 2 and 4% (*v*/*v*) EO in 99.8% methanol on *A. artemisiifolia* axillary buds (about 1 cm high) grown in vitro. 50 µL applied on filter paper placed on a metal holder stuck into the culture medium in a 350 mL glass jar.	Inhibition of shoot and root growth of *A. artemisiifolia*: 64 and 65% inhibition of shoot fresh weight with 4% *N. rtanjensis* EO and *N. cataria* EO, respectively; 100 and 80% inhibition of root fresh weight with 4% *N. rtanjensis* EO and *N. cataria* EO, respectively. Discoloration of the shoot. Suppression of CAT and SOD activity and stimulation of POX activity with both EOs tested. *N. cataria* EO had a stronger effect on CAT and POX activity, *N. rtanjensis* EO had a stronger effect on SOD activity.	[37]
*N. rtanjensis* Diklić et Milojević	*trans,cis*-Nepetalactone (72%) *cis,trans*-Nepetalactone (16%) *α*-pinene (3%)				
*N. curviflora* Boiss. collected in North Lebanon	*β*-Caryophyllene (41.6%) Caryophyllene oxide (9.5%) *trans-β*-Farnesene (6.2%) *cis-β*-Farnesene (4.8%)	*Lepidium sativum* L. *Raphanus sativus* L.	In vitro assay in Petri dishes on filter paper. Dose tested: 2.5, 1.25, 0.625, and 0.25 mg EO mL^−1^ water–acetone mixture (99.5:0.5).	No effect on seed germination. Inhibition (49 and 42%) of *R. sativus* radicle length with *N. nuda* subsp. *albiflora*—1800 masl EO at concentrations of 1.25 and 2.5 mg mL^−1^, respectively.	[98]
*N. nuda* L. subsp. *albiflora* (Boiss.) Gams. collected in Mount Lebanon at 1400 masl	*β*-Bisabolene (11.8%) Pulegone (10.8%) *trans,cis*-Nepetalactone (8.0%) *trans-β*-Farnesene (7.1%) Caryophyllene oxide (6.9%)				
*N. nuda* L. subsp. *albiflora* (Boiss.) Gams. collected in North Lebanon at 1800 masl	Hexadecanoic acid (10.1%) *β*-Bisabolene (7.8%) Caryophyllene oxide (7.3%) Pulegone (7.2%) *trans,cis*-Nepetalactone (4.4%)				
*N. x faassenii* Bergmans ex Stearn	*cis,trans*-Nepetalactone (73.05%) germacrene B (10.25%) *trans,cis*-Nepetalactone (5.79%)	*Lepidium sativum* L.	In vitro enclosed volatile bioassays on filter paper. Dose tested: 5, 10, and 20 g of leaves in cheesecloth per 500 mL glass flask.	Inhibition of shoot growth (21 and 48%) at 5 g and 10 g of foliage, respectively. Inhibition of root elongation (7 and 44%) at 5 g and 10 g of foliage, respectively. Inhibition of radicle elongation and shoot growth (100%, both) at 20 g of foliage.	[99]
*N. flavida*	Linalool (37.64%) 1,8-Cineole (30.80%)	*Eruca sativa* Mill. *Lepidium sativum* L. *Raphanus sativus* L.	In vitro assay in Petri dishes on filter paper. Dose tested: 0.25, 0.5, 1.0, 2.0, 4.0, and 8.0 μL EO mL^−1^ Tween 80–water solution (0.5%, *v*/*v*).	Inhibition of seed germination (100%) at 4 μL and 8 μL EO mL^−1^ in all tested weeds. Inhibition of seed germination (84, 83, and 75%) at 2 μL EO mL^−1^ in *E. sativa*, *R. sativus*, and *L. sativum*, respectively. Inhibition of radicle and plumule length of *R. sativus* (93 and 91%), *L. sativum* (89 and 88%), and *E. sativa* (93 and 97%) at 2 μL EO mL^−1^.	[100]
*N. glocephalata* Rech.f	1,8-Cineole (34.1%) *β*-Pinene (21.5%) *α*-Pinene (8.1%) Sabinene (7.8%) (Z)-*β*-Ocimene (7.6%) (E)-*β*-Ocimene (6.9%)	*Amaranthus retroflexus* L. *Chenopodium album* L. *Echinochloa crus-galli* (L.) Beauv *Phalaris canariensis* L.	In vitro assay in Petri dishes on filter paper. Dose tested: 1, 2, 4, and 8 μL EO mL^−1^ water solution. In a greenhouse assay, foliar application: 1.25, 2.5, 5, and 10% EO in water (*v*/*v*) on 3-week-old plants.	Inhibition of seed germination and root and shoot length by increasing the concentration of the tested EOs. *A. retroflexus* and *C. album* were most sensitive to *N. isphanica* EO and *N. glocephalata* EO: 100% inhibition of seed germination at 4 μg *N. isphanica* EO/mL and 100% inhibition at 8 μg *N. glocephalata* EO mL^−1^. *N. ispahanica* EO inhibited the root length of all tested weeds more strongly than *N. glocephalata* EO. Shoot length was less affected in *N. isphanica* EO and *N. glocephalata* EO than the root length of all tested weeds. Chlorosis to necrosis of the weeds 7 days after spraying. Reduction in the dry mass of the seedlings and the Chl a and Chl b content 7 days after spraying. About 10% higher inhibition of dry mass and about 5% higher inhibition of Chl a and Chl b content with *N. isphanica* EO (10%) compared to *N. glocephalata* EO (10%). Monocotyledonous weeds were more resistant than the dicotyledonous weeds tested.	[96]
*N. ispahanica* Boiss	1,8-Cineole (66.4%) *β*-Pinene (10.7%) *α*-Pinene (3.1%)				
*N. meyeri* Benth.	*trans,cis*-Nepetalactone (80.3%) *cis,trans*-Nepetalactone (10.3%) *trans*-Pulegol (3.1%) 1,8-Cineole (3.0%) *β*-Bourbonene (2.0%)	*Amaranthus retroflexus* L. *Chenopodium album* L. *Cirsium arvense* (L.) Scop. *Sinapis arvensis* L.	In vitro assay in Petri dishes on filter paper. Dose tested: 0.5, 1.0, and 2 mg µL^−1^ DMSO–water solution (10%, *v*/*v*). In a greenhouse assay, spraying weeds at the 3–4 leaves stage with 20 mg EO mL^−1^ of DMSO–water solution (10%, *v*/*v*).	Inhibition of seed germination of *A. retroflexus*, *C. album*, *C. arvense*, and *S. arvensis* (100%) at all tested concentrations. Mortality of plants from 53.33 ± 6.36% (*S. arvensis*) to 64.00 ± 5.29% (*A. retroflexus*) 48h after treatments with 20 mg EO mL^−1^.	[93]
*N. meyeri* Benth.	*trans,cis*-Nepetalactone (83.4%) *cis,trans*-Nepetalactone (8.83%)	*Amaranthus retroflexus* L. *Bromus danthoniae* Trin. *Bromus intermedius* Guss. *Chenopodium album* L. *Cynodon dactylon* L. *Lactuca serriola* L. *Portulaca oleracea* L.	In vitro assay in Petri dishes on filter paper. Dose tested: 0.01 and 0.02% EO in Tween 20–water solution (0.01%, *v*/*v*).	Inhibition of seed germination (100%) at 0.02% EO in *A. retroflexus*, *B. danthoniae*, *B. intermedius*, and *L. serriola*. Inhibition of seed germination (more than 70%) at 0.02% EO in *C. album* and *C. dactylon*. CAT activity increased and SOD activity decreased in all weed species except *A. retroflexus*. H_2_O_2_ concentration and level of lipid peroxidation increased in all tested weeds.	[95]
*N. nervosa* Royle & Bentham.	Nepetalactone (a very low concentration)	*Lepidium sativum* L.	In vitro enclosed volatile bioassays—seed germination and growth of seedlings, as well as activities of antioxidant enzymes. Cocultivation of 1, 3, 5, 7, and 9 4-week-old plants of *N. rtanjensis*, *N. sibirica*, and *N. nervosa* with *L. sativum* seeds in a 350 mL glass vessel with culture medium for 5 days.	Inhibition of seed germination and seedling growth when co-cultured with *N. rtanjensis* and *N. sibirica*. *N. rtanjensis* had stronger effects than *N. sibirica*. No effect on seed germination when co-cultured with *N. nervosa*. Inhibition of CAT and POX activity and changes in the profiles of Fe- and Cu/Zn- isoforms of SOD when co-cultured with *N. rtanjensis* and *N. sibirica*.	[35,36]
*N. rtanjensis* Diklić et Milojević	*trans,cis*-Nepetalactone (5–23 ppbV)				
*N. sibirica* L.	*cis,trans*-Nepetalactone (3–25 ppbV)				
*N. nuda* L. subsp. *albiflora*	*trans,cis*-Nepetalactone (74.27%) 2(1H)-Naphthalenone, octahydro8a-methyl-trans- (10.09%)	*Lactuca sativa* L. *Lepidium sativum* L. *Portulaca oleracea* L. *Raphanus sativus* L. *Triticum aestivum* L.	In vitro assay in Petri dishes on filter paper. Dose tested: 0.015, 0.031, 0.062, 0.125, 0.25, 0.5, 1, and 2 μL EO mL^−1^ Tween 80–water solution (0.5%, *v*/*v*)	Inhibition of seed germination of *T. aestivum*, *L. sativa*, *R. sativus*, and *L. sativum* (100%) at 0.5 µL EO mL^−1^ and at higher concentrations. Inhibition of radicle and pulmule growth of *R. sativus* (88 and 74%), *L. sativa* (81 and 86%), *T. aestivum* (75 and 73%), *P. oleracea* (71 and 64%), and *L. sativum* (56 and 64%), respectively, at 0.25 μL EO mL^−1^. Inhibition of fresh and dry weight of *T. aestivum* (85 and 82%), *L. sativa* (77 and 84%), *R. sativus* (70 and 70%), *L. sativum* (66 and 47%), and *P. oleracea* (53 and 41%) at 0.25 μL EO mL^−1^.	[101]
*N. pannonica* L.	1,8-Cineole (28.9%) *trans,cis*-Nepetalactone (14.3%)	*Agrostis stolonifera* L. cv. Pencross *Lactuca sativa* L. cv. Iceberg	In vitro assay in 24-well plates. Dose tested: 0.01, 0,03, 0,1, 0.3, and 1 mg EO mL^−1^ water; 2 mg *trans,cis*-nepetalactone mL^−1^ water.	Inhibition of growth of *A. stolonifera* (100%) at 0.3 and 1.0 mg EO mL^−1^. No effect on seed germination at 2 mg *trans,cis*-nepetalactone mL^−1^.	[102]
*N. rtanjensis* Diklić et Milojević	*trans,cis*-Nepetalactone (72%) *cis,trans*-Nepetalactone (16%) *α*-Pinene (3%) *β*-Pinene (0.4%)	*Arabidopsis thaliana* (L.) Heynh. *Brassica napus* L. cv. *napus* *Lactuca sativa* L. cv. May Queen *Lepidium sativum* L. *Lotus corniculatus* L. cv. Bokor *Rumex crispus* L. *Stellaria media* (L.) Vill.	In vitro enclosed volatile bioassays in Petri dishes—seed germination. Dose tested: 0.1, 0.3, 0.5, 1, 2, and 4% of *N. rtanjensis* EO, *α*-pinene, and *β*-pinene in 99.8% methanol on seeds germinated on culture media. 30 µL of *N. rtanjensis* EO, *α*-pinene, and *β*-pinene applied to filter paper in Petri dishes that are not in direct contact with the culture medium.	Among the crop plants, *L. sativa* was the most sensitive, while *B. napus* showed the highest tolerance to *N. rtanjensis* EO and *α*- and *β*-pinene. Among the weeds, *S. media* was the most sensitive, while *R. crispus* was the most tolerant to *N. rtanjensis* EO and *α*- and *β*-pinene. *α*- and *β*-pinene exhibited lower phytotoxicity than *N. rtanjensis* EO. *α*-Pinene exhibited greater phytotoxicity than *β*-pinene. *α*-Pinene particularly affected the seed germination of *L. sativa*, *L. corniculatus*, *B. napus*, and *S. media*.	[38]
*N. rtanjensis* Diklić et Milojević	Nepetalactones (*cis,trans*- and *trans,cis*) with [M+1]^+^ at *m*/*z* 167 *α*- and *β*-Pinene with [M+1]^+^ at *m*/*z* 137 *α*-campholenal, neral, and geranial with [M+1]^+^ at *m*/*z* 153 *γ*-cadinene, *δ*-cadinene, *cis*- and *trans*-caryophyllene, and *α*-humulene with [M+1]^+^ at *m*/*z* 205	*Arabidopsis thaliana* (L.) Heynh	In vitro enclosed volatile bioassays—plant growth, Chl, organic acid, sugar and ammonium levels, activities and abundance of GS and antioxidant enzymes, and expression of GS-encoding genes were investigated. Dose tested: 2 and 4% (*v*/*v*) EO in 99.8% methanol on 2-week-old *A. thaliana* plants grown in vitro. 50 µL of EO was applied to filter paper placed on a metal holder in the culture medium in 350 mL glass vessels. Nepetalactone concentrations at the beginning of the experiment: 1700 and 3400 ppbV in the atmosphere of the glass vessels with 2 and 4% EO, respectively. Nepetalactone concentration after 10 days: 18 ppbV and 29 ppbV in the atmosphere of the glass vessels with 2 and 4% EO, respectively. The same trend was observed for other traced compounds.	No effect on the Chl content (Chl a, Chl b, and Chl a+b) or the fresh weight of the roots. Decrease in fresh weight of shoots at higher EO concentrations (4%). Increase in the content of isocitric acid, malic acid, and succinic acid with a simultaneous decrease in the fructose and sucrose content in the shoots. No effects on GS activity, GS abundance, and ammonium content in the shoots. Changes in the activities of antioxidant enzymes: CAT activity decreased in both shoots and roots, with more pronounced effects in shoots, POX activity decreased in the roots, SOD remained unchanged in the shoots but showed a dose-dependent response in the roots, decreasing at lower EO concentrations and increasing at higher EO concentrations. No changes in the expression of *GLN1;1*, *GLN1;2*, *GLN1;3*, *GLN1;4*, and *GLN2* in *A. thaliana* shoots.	[40]
*N. rtanjensis* Diklić et Milojević	*trans,cis*-Nepetalactone (72%) *cis,trans*-Nepetalactone (16%) *α*-pinene (3%)	*Amaranthus retroflexus* L. *Ambrosia artemisiifolia* L. *Artemisia vulgaris* L. *Cephalaria transsylvanica* (L.) Schrad. ex Roem. & Schult *Stellaria media* (L.) Vill.	In vitro assay in Petri dishes on filter paper. Dose tested: 0.006, 0.013, 0.025, 0.05, and 0.1% of the EO for *A. retroflexus*, *A. artemisiifolia*, and *C. transsylvanica*, 0.003, 0.006, 0.013, 0.025, 0.05, and 0.1% of the EO for *A. vulgaris* and *S. media*, respectively. EO:methanol:Tween 20 in a volume ratio of 1:4:1 and water was added to the desired EO concentration. In the greenhouse assay, the soil was sprayed with EO (0.06, 0.13, 0.25, 0.5, and 1%) before sowing *S. media* seeds, and the *S. media* seedlings at the 2-node stage were sprayed with EO (0.06, 0.13, 0.25, 0.5, and 1%) 12 days after sowing. EO:methanol:Tween 20 at a volume ratio of 1:1:1 and water was added to the desired EO concentration.	Inhibition of seed germination of *S. media* (100%) at EO (from 0.013 to 0.1%). Inhibition of seed germination of *A. retroflexus*, *A. vulgaris*, and *A. artemisiifolia* (100, 100, and 48%, respectively) at EO (0.1%). Foliar spraying of *S. media* seedlings 12 days after sowing with EO (0.5 and 1%) resulted in a significant reduction in shoot height, fresh weight, and node formation. Mortality of *S. media* seedlings (45%) 5 days after spraying with EO (1%).	[41]
*N. rtanjensis* Diklić et Milojević	*trans,cis*-Nepetalactone (72%) *cis,trans*-Nepetalactone (16%)	*Arabidopsis thaliana* (L.) Heynh	In a greenhouse assay, foliar application of 10 μL of 2% EO on 10 leaves of 4-week-old *A. thaliana* plants	Leaf discoloration and a reduction in the fresh weight of the shoots 10 days after treatment. Chl a, Chl b, and Chl a+b content decreased slightly. Change of organic acids and reduction of glucose and fructose content in the shoots of *A. thaliana* 10 days after treatment. Slight increase in total GS activity without changes in ammonia levels 1 and 10 days after treatments. Upregulation of the expression of GS-encoding genes, *GLN1;1* and *GLN1;3*, 1 day after treatment.	[39]

## Data Availability

Data is contained within the article.

## Abbreviations

The following abbreviations are used in this manuscript:

ABA	Abscisic acid
ABC	ATP-binding cassette
ARD	Apple replant disease
ATP	Adenosine triphosphate
CAT	Catalase
CDK	Cyclin-dependent kinase
Chl	Chlorophyll
CMPG	Carboxymethylation
CYC	Cyclin
DHC	Dihydrochalcones
DHNL	Dihydronepetalactone
DMBA	7,12-dimethylbenz[a]anthracene
DMAPP	Dimethylallyl diphosphate
DPPH	1,1-diphenyl-2-picryl-hydrazyl
EO	Essential oil
FDA	Food and Drug Administration
FGPP	Farnesyl geranyl pyrophosphate
FGPPS	Farnesyl geranyl pyrophosphate synthase
FPP	Farnesyl pyrophosphate
FPPS	Farnesyl pyrophosphate synthase
G8H	Geraniol 8-hydroxylase
GES	Geraniol synthase
GGPPS	Geranylgeranyl pyrophosphate synthase
GPP	Geranyl pyrophosphate
GPPS	Geranyl pyrophosphate synthase
GS	Glutamine synthetase
H_2_O_2_	Hydrogen peroxide
HGO	Hydroxygeraniol oxidase
IAA	Indole-3-acetic acid
IPP	Isopentenyl diphosphate
ISY	Iridoid synthase
KEGG	Kyoto encyclopedia of genes and genomes
MDR/PGP	Multi-drug resistance/P-glycoprotein
MEP	2-C-methyl-D-erythritol 4-phosphate
MLPL	Major-latex protein-like
MVA	Mevalonate
NA	Nepetalic acid
NADH	Nicotinamide adenine dinucleotide hydrogen
NE	Nanoemulsion
NL	Nepetalactone
NLC	Nanostructured lipid carriers
*Nc*EO	*Nepeta cataria* EO
*Nr*EO	*Nepeta rtanjensis* EO
^1^O_2_	Singlet oxygen
O_2_⁻	Superoxide radicals
OH^•^	Hydroxyl radicals
OxIAA	Oxindole-3-acetic acid
PAT	Polar auxin transport
PEPC	Phosphoenolpyruvate carboxylase
PIN	Pin-formed
POX	Peroxidase
PPT	Phosphinothricin
ROS	Reactive oxygen species
SDR	Short-chain dehydrogenase
SLN	Solid lipid nanoparticle
SOD	Superoxide dismutase
UHPLC	Ultra-high-performance liquid chromatography
VOC	Volatile organic compound
WE	Water extract
